# Noncovalently bound excited-state dimers: a perspective on current time-dependent density functional theory approaches applied to aromatic excimer models†

**DOI:** 10.1039/d3ra07381e

**Published:** 2023-12-11

**Authors:** Amy C. Hancock, Lars Goerigk

**Affiliations:** a School of Chemistry, The University of Melbourne Parkville Australia lars.goerigk@unimelb.edu.au +61-(0)3-8344 6784

## Abstract

Excimers are supramolecular systems whose binding strength is influenced by many factors that are ongoing challenges for computational methods, such as charge transfer, exciton coupling, and London dispersion interactions. Treating the various intricacies of excimer binding at an adequate level is expected to be particularly challenging for time-dependent Density Functional Theory (TD-DFT) methods. In addition to well-known limitations for some TD-DFT methods in the description of charge transfer or exciton coupling, the inherent London dispersion problem from ground-state DFT translates to TD-DFT. While techniques to appropriately treat dispersion in DFT are well-developed for electronic ground states, these dispersion corrections remain largely untested for excited states. Herein, we aim to shed light on current TD-DFT methods, including some of the newest developments. The binding of four model excimers is studied across nine density functionals with and without the application of additive dispersion corrections against a wave function reference of SCS-CC2/CBS(3,4) quality, which approximates select CCSDR(3)/CBS data adequately. To our knowledge, this is the first study that presents single-reference wave function dissociation curves at the complete basis set level for the assessed model systems. It is also the first time range-separated double-hybrid density functionals are applied to excimers. In fact, those functionals turn out to be the most promising for the description of excimer binding followed by global double hybrids. Range-separated and global hybrids—particularly with large fractions of Fock exchange—are outperformed by double hybrids and yield worse dissociation energies and inter-molecular equilibrium distances. The deviation between each assessed functional and reference increases with system size, most likely due to missing dispersion interactions. Additive dispersion corrections of the DFT-D3(BJ) and DFT-D4 types reduce the average errors for TD-DFT methods but do so inconsistently and therefore do not offer a black-box solution in their ground-state parametrised form. The lack of appropriate description of dispersion effects for TD-DFT methods is likely hindering the practical application of the herein identified more efficient methods. Dispersion corrections parametrised for excited states appear to be an important next step to improve the applicability of TD-DFT methods and we hope that our work assists with the future development of such corrections.

## Abstract 2

We assess most recent TD-DFT methods on excimers and highlight shortcomings of current strategies to treat dispersion in excited states.

## Graphical abstract

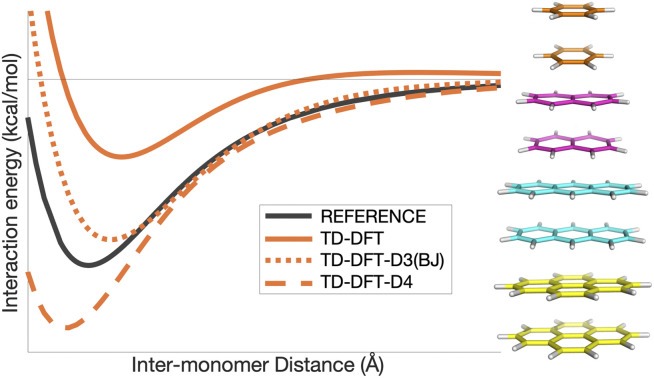

## 1 Introduction

Exciplexes are short-lived, heterodimeric “excited complexes” that are stable in the electronic excited state while dissociative in the electronic ground state.^1–3^ Their formation can be considered as the association between an excited monomer 
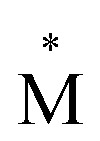
 and a second monomer N in the ground state, as depicted by the following reaction scheme:1
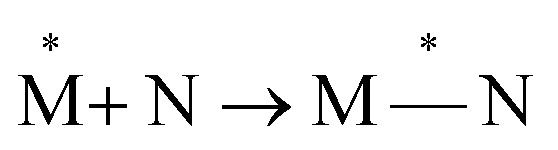
where the asterisk denotes an electronically excited species. Ideally, the monomers are polar or polarisable species able to facilitate attractive charge-transfer (CT) interactions in an excited state.^1^ In the homodimeric case (M = N), exciplexes are called “excimers”, which are the focus of this work.

**Fig. 1:**
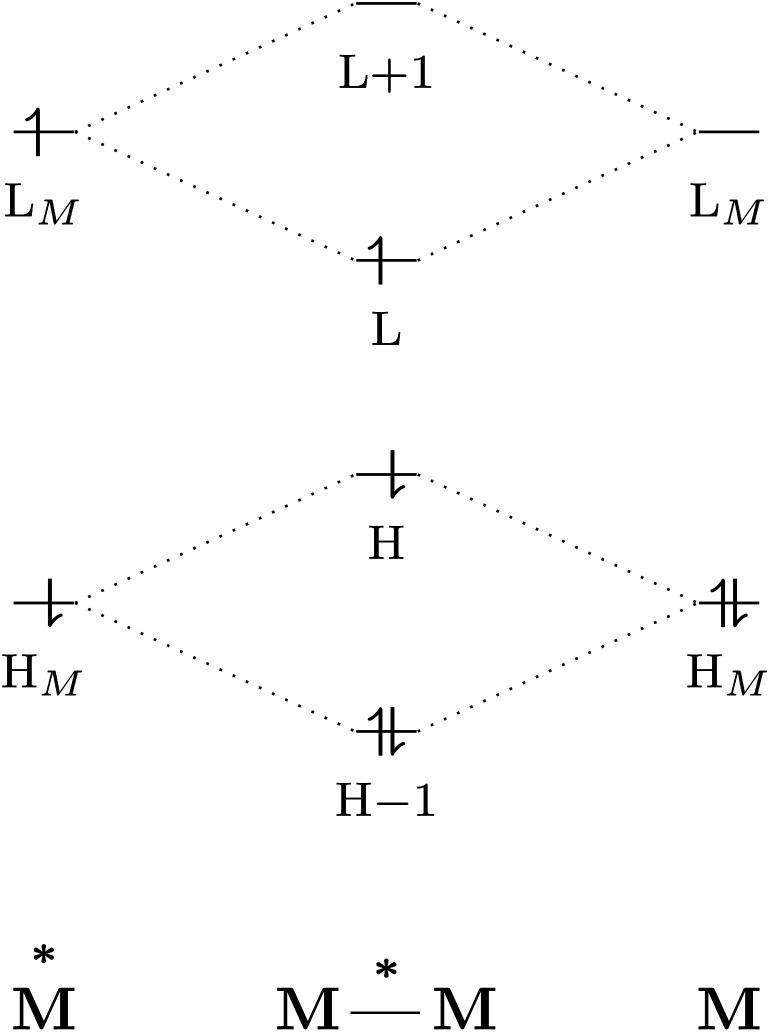
Simplified molecular orbital diagram describing the formation of an excimer from molecular orbitals of the monomers as described in eqn (1). “H” and “L” refer to the highest occupied and lowest unoccupied molecular orbitals, respectively; based on similar examples in ref. 1 and 5–7.

The excited-state properties of the dimeric species are unique from those of either monomer.^4^ The stabilising effect in exciplexes and excimers can be rationalised with molecular orbital (MO) theory and a collision model of the reaction in eqn (1). When the two monomers collide, the predominant interactions occur between the highest occupied (HOMO) and lowest unoccupied (LUMO) molecular orbitals of each species to form a new set of orbitals as shown in the simplified diagram in Fig. 1.^1,5–7^ In the case of two ground-state molecules, the resulting constructive and destructive-interference contributions cancel, resulting in minimal to no net stabilisation. In an excimer or exciplex, the electronically excited species causes two interacting orbitals to be singly occupied resulting in stabilisation through formation of the complex.^5^ This electronic stabilisation effect is short-lived, as relaxation to the ground state occurs quickly, causing repulsive intermolecular forces to oppose the relatively weak attractive forces. Energy decomposition analysis of the excimer/exciplex stabilisation energy revealed the following contributing components: electrostatics, Pauli repulsion, CT, exciton coupling and London dispersion.^8,9^

Aromatic excimers were discovered experimentally in the fluorescence spectrum of pyrene in cyclohexane solution by a broad, structureless emission band that occurred at a lower energy than the associated monomer emission.^10^ Since then, excimers have also been revealed to be crucial species in contemporary applications of technological and biological relevance. Contrary to their notoriety as an undesired energy trap for singlet-fission,^11^ some studies have shown potential advantages in organic electronics, such as excimer states mediating intramolecular electron transfer^12^ and charge separation^13^ or broadening the emission for white organic light emitting diodes;^14^ see ref. 15 for a review. Additionally, excimers have applications as chemosensors,^16,17^ molecular rulers,^18,19^ and industrial-scale lasers.^20^ Excimers also occur in biological systems, playing a role in the photo-damage of DNA as they can occur between nucleobases such as adenine^21^ and guanine.^22^

Computational chemistry techniques, particularly Kohn-Sham Density Functional Theory^23,24^ (DFT) approaches, also called density functional approximations (DFAs), are frequently used in applications of technological relevance, including the description of exciplexes and excimers. In order to have predictive character, DFAs must be robust; *i.e.*, they must yield results with equal accuracy or error margins across a variety of different chemical problems. Comprehensive benchmark studies have identified such generally applicable DFT approaches for ground-state problems including for thermochemistry, kinetics, and geometries, to name a few examples.^25–31^ In all studies, it was evident that the accurate treatment of noncovalent interactions (NCIs), in both inter- and intramolecular cases, is crucial to achieve the desired accuracy and robustness of a method. In the context of DFT, it is particularly important to properly address the correct treatment of London dispersion effects. It has been known since the mid-1990s that conventional DFAs do not capture those effects correctly.^32–35^ This sparked the development of various dispersion-corrected DFT techniques, some of which later turned out to be less effective than initially claimed,^36–38^ and others having now become the recommended default for ground-state DFT applications.^39,40^ We refer interested readers to recently published reviews^41,42^ directed at users that are unfamiliar with the field of dispersion-corrected DFT applications for ground-state problems as well as reviews that recommend the current best practice in the field,^43–45^ which includes dispersion-corrected double-hybrid^46,47^ DFAs (DHDFAs) when feasible.

The binding of excimers and exciplexes inherently relies on the system's excited-state properties and is dominated by NCIs, which introduces additional complexity compared to the ground-state case. Linear-response time-dependent DFT within the adiabatic approximation^48–50^ (TD-DFT) has become the method of choice to treat excited-state problems. Recent advances^51–53^ and detailed benchmark studies^54–64^ on single-molecule cases have shed light on the quality of TD-DFAs for the calculation of excitation energies. As recently summarised for readers unfamiliar with the field, lower rungs on the Jacob's Ladder of DFT^65^ should be avoided due to the emergence of artificial ghost states and large red shifts in excitation energies.^66^ Global-hybrid DFAs can be suitable for local valence excitations, subject to having the right amount of nonlocal Fock exchange (FE)—about 40% (ref. 57 and 66)—but fail for CT and other long-range transitions.^52,57,62,67–71^ While the range-separation (RS) technique^72–75^ applied to the exchange part of DFAs solves the long-range problem, local valence excitations tend to be blue-shifted.^52,58,62,66,76^ Time-dependent DHDFAs^77^ depend additionally on a nonlocal, perturbative electron-correlation component^78^ that compensates for many of the systematic errors of hybrids.^47,51,54,55,57,58,66,79^ In fact, our group recently developed RS-DHDFAs^52,53^ that belong to some of the currently most robust TD-DFT methods for a variety of different local and long-range valence excitations in organic molecules.^52,53,62–64^ A slightly different group of RS-DHDFAs have also proven to be very promising when used within the Tamm–Dancoff Approximation^80^ (TDA-DFT).^81–83^

Compared to single-molecule cases, there are fewer systematic studies that explore NCIs in excited states of molecular complexes. However, it is expected that TD-DFT methods inherit the problems of the underlying ground-state DFA, such as the inability to properly describe London dispersion interactions. Additionally, the TD-DFT formalism itself carries problems that may further complicate the treatment of excited-state NCIs. In 2008, Huenerbein and Grimme presented a study of excimers and one exciplex across a very limited number of TD-DFAs.^5^ The study was unique in the sense that a London dispersion correction was rigorously applied to an excited-state study, namely the DFT-D2 (ref. 84) correction developed in 2006. Although the dispersion coefficients used in DFT-D2 were based on ground-state polarisabilities, the combination of DFT-D2 with high-FE global-hybrid DFAs, showed a qualitatively better agreement with experimental data compared to dispersion-uncorrected TD-DFT, both for the stability of the dimers and the inter-monomer equilibrium distance. The authors' argument was that a ground-state based correction should be seen as a lower bound for the dispersion energy in an excited-state system, as C_6_ coefficients are different, and most likely larger, for more polarisable excited states. Some of the subsequent TD-DFT based studies on excimer and exciplex systems either do not explore the dispersion problem of DFT^85,86^ or erroneously justified the use of Minnesota functionals^87^ for consideration of dispersion effects,^22,88^ despite ground-state based studies that disprove claims they are able to treat such interactions.^25,27,37,38,89^ Some other studies recognised the dispersion problem, but for a lack of a better choice, followed Huenerbein and Grimme's recommendation to use a global-hybrid combined with DFT-D2,^6,90–95^ while fewer applied the newer, ground-state based DFT-D3 method in its zero-damping^96^ [DFT-D3(0)] or in its Becke–Johnson-damping^97–99^ [DFT-D3(BJ)^40^] form.^100–106^ To our knowledge, only three studies have considered adjusting dispersion corrections for excited states. In the first, Ikabata and Nakai^107^ calculated excited-state dispersion coefficients within the local-response dispersion^108,109^ (LRD) model to explore the interaction energies of exciton-localised molecular complexes from the S66 (ref. 110) benchmark set as well as three molecular excimers. Then, Briggs and Besley^111^ empirically varied the dispersion coefficients and van der Waals radii in DFT-D2 for small model systems of ethene–argon and formaldehyde–methane complexes. And finally, Johnson and co-workers calculated dispersion coefficients within the exchange-hole dipole moment^97–99,112^ (XDM) model for excited states of conjugated hydrocarbons, pull–pull chromophores, and CT complexes.^113^ While these studies considered suitable model systems, only a limited selection of DFAs were applied. These three studies, among others,^114,115^ would allow the conclusion that state-specific dispersion corrections for excited states should be developed, but further characterisation of current methods is necessary to provide guidance towards such future developments. The present paper intends to provide such characterisation.

Most of the aforementioned exciplex studies used older global-hybrid DFAs, while some^85,86,90,91,102,105,106,116^ used range-separated hybrids. Of the two studies that investigated DHDFAs^94,102^ both utilised TDA-DFT, instead of the full TD-DFT scheme, but only Krueger and Blanquart^102^ considered a dispersion correction. Interestingly, most of the methods previously used to study exciplex systems are not those recommended by some of the latest single-molecule excitation studies,^52,53,57,62,64,66^ which by itself is reason enough to investigate the latter in the exciplex/excimer context. The majority of excimer and exciplex studies investigated these methods *via* applications,^22,86,88,91–94,100,101,104,116^ or carried out benchmark analyses against reference values that were not well defined and mostly based on experiment.^5,90^ Both benchmarks and application-based excimer studies focussed on comparison of methods by the calculation of the dissociation energy, as this explores the binding of the complex, while fewer studies have explored other spectrometric parameters such as the fluorescence, absorption and repulsion energies.^7,85,86,104,107,116–121^ Potential energy curves of the lowest excited state reported relative to the asymptote of the dissociation curve of the ground state^5^ are standard practice to derive these spectroscopic parameters. Ground-state studies commonly explore NCIs by focusing entirely on interaction energies that can be calculated, *e.g.*, as the difference between the dimer and individual monomer energies or alternatively as the difference between the total energies of the dimer and a system in which the two monomers have been dissociated to a large distance from one another. By this definition, a negative interaction energy indicates a stable complex. The dissociation energy of an excimer is inherently an interaction energy and provides a convenient metric to discuss excited-state binding and NCIs in one go. However, the computational studies of excimers and exciplexes do not report excited-state interaction energy curves, other than a handful of exceptions we are aware of;^7,8,85,102^ we advocate to adopt them more frequently, as it allows direct comparison with what has become the standard in ground-state treatments of NCIs. Note that according to that definition of an interaction energy, the dissociation energy *D*_e_ is simply its absolute value.

**Fig. 2:**
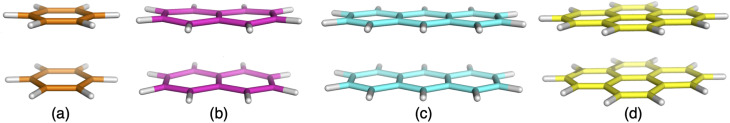
Excimer benchmark cases used in this study consisting of the following monomers: benzene (a), naphthalene (b), anthracene (c) and pyrene (d).

There have been many rigorously conducted NCI studies for ground-state systems that are usually based on a high-quality reference and compared different computational approaches on an equal footing; the benefits of using high-level computational data as opposed to experimental references has been well-established and explained elsewhere.^27,43,51,52,66^ To the best of our knowledge, similar studies are missing to systematically study NCIs in excited states. In this work, we intend to initiate the first step towards more systematic studies of excited-state NCIs by restudying excimer model systems under incorporation of the latest state-of-the art TD-DFT methods. The four model systems discussed herein are the benzene, naphthalene, anthracene and pyrene dimers (see Fig. 2) which have been studied in combination before by two prior studies.^7,90^ We discuss the change of the interaction energy for each dimer system upon dissociation, *i.e.* we discuss equilibrium and non-equilibrium geometries. In each case, we discuss the first excited state, which is the excimer state. In the following section, Section 2, we briefly outline general computational details before analysing various wave-function theory (WFT) levels based on truncated basis sets and at the complete-basis-set (CBS) limit in Section 3. The purpose of this analysis is to identify a suitable level of theory that can serve as a reliable, yet fast benchmark for the subsequent discussion of TD-DFT methods in various subsections of Section 4. The latter is split into different aspects: the impact of non-local FE, the behaviour of dispersion-uncorrected TD-DFT methods, the impact of using ground-state based corrections of the DFT-D3(BJ)^40,96^ and DFT-D4 (ref. 122 and 123) type, and an analysis of method performance averages over the four systems. In a nutshell, our study is sufficiently comprehensive to fill an important gap in our knowledge of current TD-DFT methods and additive dispersion corrections for the description of excimers.

## 2 General computational details

TURBOMOLE 7.3 (ref. 50 and 124–126) was used for geometry optimisations of the lowest-lying singlet excited states of each dimer at the spin-component-scaled^127,128^ approximate coupled cluster singles doubles (SCS-CC2 (ref. 129)) level with the def2-TZVP^130^ triple-ζ (TZ) atomic-orbital (AO) basis set and a geometry convergence criterion of 10^−7^ E_h_. Use of the RICC2 module in TURBOMOLE limits the symmetry consideration to Abelian point groups such that all excimers are calculated with *D*_2h_ symmetry. The excited-state optimised structures were used to generate non-relaxed dissociation curves as follows: the internal coordinates of each monomer were frozen, then total energies of the lowest-lying singlet excited states were calculated across a range of inter-monomer separations up to 16 Å, and then each energy was taken relative to 16 Å, which represents the asymptote for the given state. The geometry optimised excimer structures were not comprised of perfectly planar monomers, which has been previously noted during both TD-DFT and CC2 (ref. 131) optimisations of these excimers^90^ and exciplexes comprised of the same monomers.^85^ Therefore, we defined the inter-monomer separation for our dissociation curves as the distance between two central carbon atoms, opposite each other on each monomer; this parameter is visually defined for each excimer structure in Fig. S1 (ESI†). *D*_e_, is defined as the difference between the total energy of the first excited state at the 16 Å point and the total energy of the same state at the minimum point. The effect of geometric relaxation falls outside the scope of this study. However, frozen-monomer structures at the SOS-CIS(D_0_)^132^ level have been shown to give good estimates of the equilibrium inter-monomer distance, with excimer dissociation energies that are generally overestimated by *ca.* 2 kcal mol^−1^ in the case of the benzene excimer.^133^ As this study uses the same structures throughout, internal comparison of the dissociation energies is reasonably justified.

Herein, the figures display interaction energy curves such that a negative interaction energy indicates a stable dimer. In the context of this paper, *D*_e_ corresponds to the dissociation energy of an excimer and therefore the terms interaction and dissociation energy are used interchangeably. Equilibrium inter-monomer distances *r*_e_, corresponding to the distance at which the minimum energy arises, are also discussed in parts of our discussion.

TURBOMOLE was also used for all SCS-CC2 and CC2 single-point calculations along the dissociation curves. The frozen-core and resolution of the identity^134^ (RI) approximations were employed with appropriate auxiliary basis sets.^135^ Coupled cluster singles doubles with perturbative triples excitation correction^136^ [CCSDR(3)] calculations for select points along the dissociation curves were carried out with Dalton2016,^137^ also utilising the frozen-core approximation. The WFT calculations were used to identify suitable reference data for the subsequent assessment of TD-DFT methods.

Single-point, linear-response TD-DFT calculations within the adiabatic approximation were conducted for all dissociation curves for a series of DFAs as listed in Table 1. For the study of the influence of FE we apply a series of PBE^138^- and BLYP^139–141^-based methods with varying amounts of non-local exchange. However, as lower-rung DFAs have shown to be unreliable for excited-state problems,^55,66^ we limit our final benchmarking study to global hybrids, range-separated hybrids, global double hybrids and range-separated double hybrids; the exact functional types are detailed in Table 1. Those DFAs have been chosen either based on popularity or known accuracy for excited states of organic molecules; see ref. 66 for recommendations and insights. Note that time-dependent double-hybrid calculations require a conventional TD-DFT step for its hybrid portion followed by a configuration interaction singles with perturbative doubles^78^ [CIS(D)] correction as initially suggested by Grimme and Neese;^77^ see ref. 66 for a comprehensive, free-access review on this methodology and some of its latest advances.

**Table 1:** List of TD-DFT methods applied

Program	Method	Comments^a^
TURBOMOLE 7.3	BLYP,^139–141^ PBE^138^	General gradient approximation (GGA); no FE
	B3LYP,^142,143^ PBE20	Global hybrids; 20% FE
	BHLYP,^144^ PBE50	Global hybrids; 50% FE
	PBE38,^96^ BLYP38	Global hybrids; 37.5% 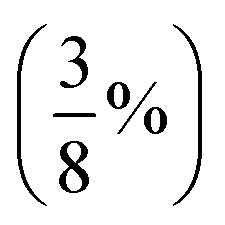 FE
	PBE75, BLYP75	Global hybrids; 75% FE
ORCA V4.1.0	CAM-B3LYP^74^	RS hybrid, but without 100% FE in the long range
	ωB97X^145^	RS hybrid with 100% FE in the long range
	B2PLYP^46^	Global double hybrid; 53% FE; 27% CIS(D) correlation
	B2GP-PLYP^146^	Global double hybrid; 65% FE; 36% CIS(D) correlation
Local version of ORCA4^b^	ωB2PLYP,^52^ ωB2GP-PLYP^52^	RS versions of B2(GP-)PLYP with 100% FE in the long range

a GGAs: rung 2; global and RS hybrids: rung 4; global and RS double hybrids: rung 5; FE: Fock exchange.

b Available in ORCA 4.2 and ORCA5.

Self-consistent-field (SCF) steps in this work were carried out with energy convergence criteria of 10^−7^ E_h_ (TURBOMOLE) and 10^−8^ E_h_ (ORCA). The frozen-core approximation was applied across all TURBOMOLE calculations, and in ORCA^147,148^ calculations it was used along with the RI approximation for the perturbative parts of the global double hybrids. Large quadrature-grid options “7” (TURBOMOLE) and “grid6 finalgrid7” (ORCA) were used to ensure smooth dissociation curves. Dispersion corrections of the type DFT-D3(BJ)^40,96^ and DFT-D4 (ref. 122 and 123) were carried out with the DFTD3 (v3.1) and DFTD4 (v2.0) standalone programs.^149,150^ Damping parameters for the various functionals were taken from the respective reference that published them first. For DFT-D3(BJ) these are ref. 40 for B3LYP, ref. 25 for PBE38, BHLYP, and CAM-B3LYP, ref. 151 for B2(GP-)PLYP, and ref. 152 for ωB2(GP-)PLYP. All DFT-D4 parameters were taken from ref. 123 except for PBE38 and the two RS-DHDFAs which were taken from ref. 152 and 153, respectively.

A series of different basis sets—ranging from double-ζ (DZ) to quadruple-ζ (QZ)—were employed throughout this work, with some of them used for extrapolations to the CBS limit. More information is provided in the following sections.

## 3 Establishing a suitable reference

Prior to analysing the performance of TD-DFT methods, a sufficiently reliable reference method that is still feasible for larger systems must be identified. An appropriate reference method should minimise the computational cost while maintaining reasonable comparability to a higher level of theory. Coupled-cluster WFT methods are considered the gold standard of chemical accuracy.^154,155^ For ground states, coupled-cluster singles-and-doubles with perturbative triples, CCSD(T),^156^ at the CBS limit is the ideal that many aim to achieve in contemporary benchmarking.^27,157,158^ It constitutes a very accurate approximation to the true interaction energy of noncovalently bound complexes in their electronic ground state.^27,158–160^ One excited-state equivalent to ground-state treatments with CCSD(T) is linear-response CCSD enhanced with a different type of perturbative triples correction than the aforementioned CCSDR(3).^136^ It delivers excitation energies that are similar to the more costly linear-response approximate coupled cluster singles doubles triples (CC3);^161^ for examples of CCSDR(3) and CC3 being established as benchmarks and comparisons between both, see ref. 51, 52 and 162–165. While the ground-state gold standard has, in recent years, become increasingly feasible for large systems of up to several hundred atoms,^166,167^ the excited-state equivalent remains prohibitively expensive.^155^ For most of our systems, CCSDR(3) is therefore not achievable for computational reasons, and in the following we identify if instead CC2 or its spin-component-scaled^128^ variant, SCS-CC2, can be used as a low-cost alternative.

Establishing a reference method also requires the careful choice of an appropriate basis set. Large basis sets quickly become computationally prohibitive, especially for large systems, while small basis sets are rife with errors due to being incomplete. Studies on obtaining vertical excitation energies, including studies on DHDFAs, have shown that large TZ basis sets are often sufficient to obtain nearly converged results, with little change when using QZ basis sets.^51,66^ That being said, as interaction energies in the excited state have not been studied frequently, little is known about the effects of truncated basis sets on interaction energies in excited states.

The basis set superposition error (BSSE), for example, plagues the treatment of ground-state NCIs with small AO basis sets in both WFT and DFT methods. This well-known error is caused by the limited number of AOs available on a molecular fragment ‘borrowing’ the basis functions from other fragments, which artificially stabilises the multi-fragment system—such as a dimer—relative to its separate fragments.^168^ Small basis sets also suffer from basis set incompleteness error (BSIE) which can artificially destabilise complexes through a failure to correctly describe intermolecular electron density.^168^ In the case of interaction energies, BSIE is not consistent with varied inter-monomer separation and will therefore not be cancelled by similar error of infinitely separated monomers in the calculation of interaction energies.^169^ If use of a larger basis set is not suitable, additive corrections have been developed for ground states to minimise the BSSE.^170–172^ BSIE corrections^173,174^ have also been developed although they are generally scarce and more computationally demanding. To our knowledge BSSE corrections have not yet been developed for excited states, and basis set convergence studies for such systems are sparse.^121,169^ Some previous exciplex studies^7,8,88,90,102,104,120,121,169^ opted to utilise the counterpoise correction^175^ to account for BSSE in exciplex binding. Our own basis set study below utilises BSSE-uncorrected interaction energies in interest of time and avoiding corrections that have not been thoroughly assessed specifically for excited states. In that way we are also able to provide a picture of current methodology that can be easily applied by method users. Moreover, we strive to use large basis sets for CBS extrapolations, which further reduces the impact of BSSE. That being said, it is worthwhile to explore the influence of counterpoise or similar corrections in later studies. A BSIE correction for excited states was recently developed Loos and co-workers that was shown to recover chemically accurate vertical excitation energies of small organic molecules with augmented double-ζ basis sets, with the exception of diffuse excited states.^176^ This suggests that work to establish basis set error corrections for excited states is underway, however, current methods do not seem to be robust enough yet to provide reliable corrections for our present study.

Note that we explore the CBS limit with extrapolation techniques that were originally developed for ground-state problems and have to our knowledge never been assessed for NCI energies in excited states.

### 3.1 Which CC2 variant is more appropriate?

For this section, non-relaxed dissociation curves for the first excited state of the benzene dimer were generated with CC2 and SCS-CC2 and various AO basis sets. While CC2 and SCS-CC2 both yield reliable excitation energies, albeit still above the related chemical-accuracy threshold of 0.1 eV,^66,177^ benchmark studies suggest that the latter does not show consistent improvement to vertical excitation energies in general.^177,178^ However, enhanced excited-state geometries and vibrational frequencies could lead to more accurate 0–0 transition energies for π → π* and n → π* excitations.^179–181^ Therefore, any potential improvements for SCS-CC2 seem to be problem specific, hence we need to carry out a comparison with CC2.

**Fig. 3:**
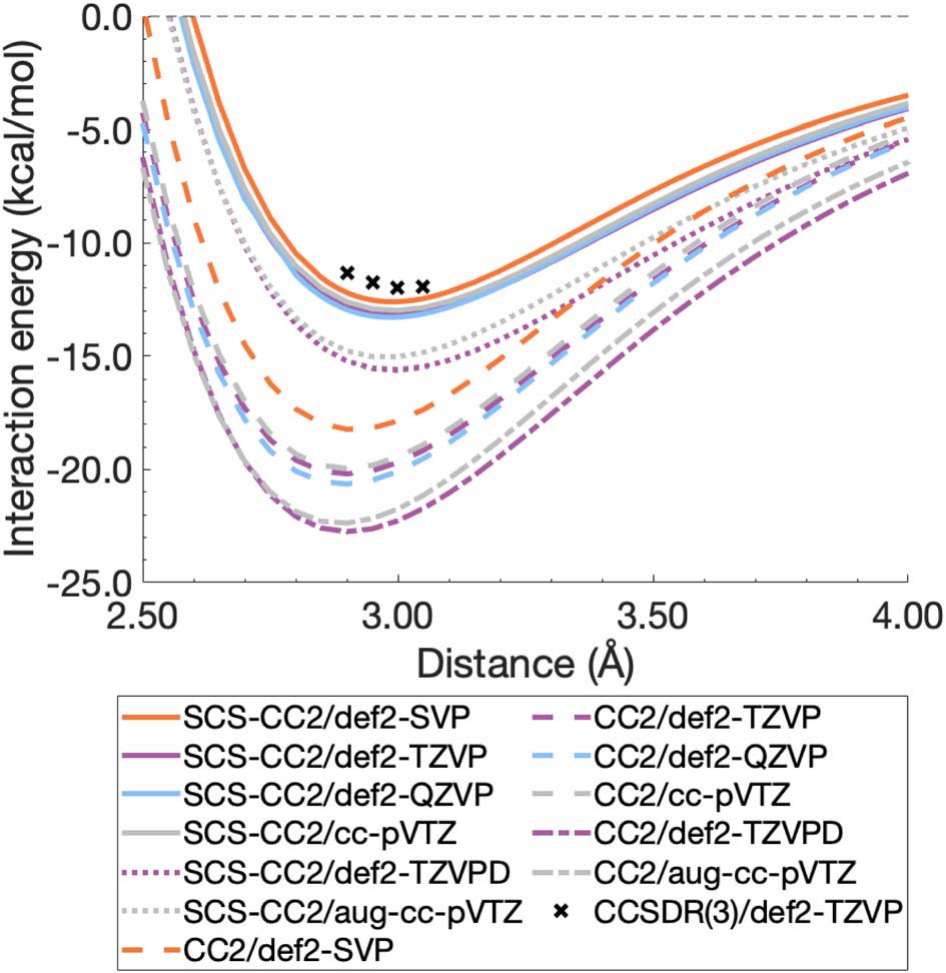
Benzene excimer dissociation curves for various CC2 and SCS-CC2 based levels of theory compared to select CCSDR(3)/def2-TZVP points.

To decide between CC2 and SCS-CC2, we conduct a study across DZ-, TZ-, and QZ-quality basis sets both with and without additional diffuse functions. This comparison involves both Ahlrichs [def2-nZVP(D)]^130,182^ and Dunning [(aug-)cc-pVnZ]^183,184^ basis sets; where n corresponds to ‘S/D’, ‘T’, ‘Q’, respectively. All dissociation curves are shown in Fig. 3 including select points for CCSDR(3)/def2-TZVP near the minimum to allow for an initial evaluation. Note that due to its computational cost, generating complete dissociation curves with CCSDR(3) was technically not feasible. Numerical values for interaction energies and intermolecular distances at the respective curve minima are shown in Table S1.†

It is obvious that all CC2 minima—regardless of the basis set—are deeper than the SCS-CC2 ones, meaning that CC2 consistently gives larger dissociation energies (Fig. 3). CC2 results indicate systematic overstabilisation, exhibiting dissociation energies that are almost twice as large as CCSDR(3). For instance, with the def2-TZVP basis set, the CCSDR(3) interaction energy at an intermolecular distance *r* of 3.00 Å (a point in proximity of the expected minimum for this level of theory) is −11.96 kcal mol^−1^ (Table S2†) and the well depths of SCS-CC2 and CC2 are −13.18 kcal mol^−1^ (*r*_e_ = 2.99 Å) and −20.19 kcal mol^−1^ (*r*_e_ = 2.90 Å), respectively. While the differences between CC2 and SCS-CC2 are quite striking, the general trends show parallels to ground-state studies, where it has been established that conventional MP2 overestimates interaction energies in dispersion-driven complexes, while SCS-MP2 provides a more balanced description.^28,128^ CC2 has also been shown to overestimate the dispersion contribution in the same excimer complexes as studied here^90^ and it appears, by its closer proximity to CCSDR(3), that SCS-CC2 reduces this overestimation. Despite SCS-CC2 being in closer proximity to CCSDR(3), both CC2 variants across all basis sets explored overstabilise the excimer relative to CCSDR(3)/def2-TZVP. When inspecting Fig. 3, we observe that the well depth increases for both CC2 variants with increasing cardinal number. For instance, we observe a change in interaction energy from −12.60 kcal mol^−1^ (def2-SVP) to −13.29 kcal mol^−1^ (def2-QZVP) for SCS-CC2, and from −18.22 (def2-SVP) to −20.64 kcal mol^−1^ (def2-QZVP) for CC2 (Table S1†).

Basis sets including diffuse functions—aug-cc-pVTZ and def2-TZVPD—produce minima that are considerably lower than basis sets without diffuse functions, meaning that the absolute interaction energies are larger for both CC2 variants in those cases. As CC2 more greatly overestimates the interaction energy minima, additional diffuse functions give well depths furthest from the CCSDR(3) reference. Interestingly, def2-TZVPD produces a deeper minimum than aug-cc-pVTZ. For SCS-CC2, def2-TZVPD and aug-cc-pVTZ differ by 0.58 kcal mol^−1^, while for CC2 they differ by 0.38 kcal mol^−1^. Without diffuse functions, Dunning and Ahlrichs basis sets are closer in energy; for instance, def2-TZVP and cc-pVTZ minima differ by 0.19 kcal mol^−1^ and 0.24 kcal mol^−1^ for SCS-CC2 and CC2 respectively (Table S1†). The choice to include diffuse functions is highly system and method dependent as in some cases their addition can increase the BSSE of the system.^185^ In the case of exciplex binding, diffuse functions giving a worse result may suggest that the BSSE and BSIE do not decrease at the same rate.^169^ The impact of diffuse functions is of interest for future studies. Herein, we choose to discard them for pragmatic reasons, as the CCSDR(3) data could not be obtained with diffuse functions. However, this decision will not influence our subsequent TD-DFT benchmark study, as long as the same type of basis set is used therein. Our final findings and conclusions are therefore unlikely to be affected by this decision.

Based on the herein discussed findings, we rule out using the CC2 method in the remainder of the study due to its greater tendency to overestimate the well depths relative to CCSDR(3). Interaction-energy curves for Ahlrichs and Dunning basis sets without diffuse functions, show reasonable agreement for SCS-CC2, with the def2-TZVP wellbeing only by 0.19 kcal mol^−1^ deeper than for cc-pVTZ. Given that Ahlrichs basis sets are computationally more efficient due to relying on fewer primitive Gaussian-type orbitals, they are our preference in this study, particularly when considering the larger dimers. In order to further clarify the best choice of basis set for SCS-CC2 as a wave function reference, CBS values are generated for CCSDR(3) and SCS-CC2 in the following section.

### 3.2 Complete basis set extrapolations

The total energy calculated by a given method is expected to converge to a finite value with an increase in AO basis set size. This also leads to converging interaction energies, as indicated by the series of interaction energies ranging from DZ to QZ basis sets discussed in the previous section, with changes between TZ and QZ being smaller than between DZ and TZ. CBS extrapolations take advantage of this convergence behaviour to estimate the fully converged energy for a given family of basis sets. While the practice of CBS extrapolation is well established for ground-state studies, CBS extrapolations are scarcely conducted for excited-state problems due to a lack of established extrapolation methods. Prior to the development of established excited-state extrapolation methods, application of ground-state extrapolations should at least improve the ground state at the base of excited-state energies. This improvement of the ground-state energy is expected to extrapolate the convergence behaviour enough to give us further insight into the best comparison of SCS-CC2 to CCSDR(3). We therefore chose to employ the same extrapolation formulae and the same extrapolation exponents as in ground-state studies. Further studies to comprehensively investigate basis set convergence behaviour of NCIs in excited states, such as that conducted by Krueger and Blanquart for exciplex systems,^169^ are strongly encouraged but such characterisation falls outside the scope of this study.

In ground-state extrapolations the HF and correlation energies are extrapolated separately due to their different convergence behaviours. The equivalent to ground-state HF energy in excited-state coupled-cluster treatments is the CCS total energy as it does not include electron correlation. We conducted linear-response calculations, so CCS total energies (*E*_CCS_) were obtained by adding the CCS excitation energy to the HF ground-state total energy. Such excited-state total energies for two truncated basis sets were then used to obtain the resulting CBS-limit energy with the familiar formula:^186^2
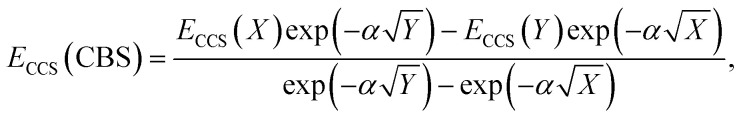
where *X* and *Y* are the successive cardinal numbers of the two basis sets used, and *α* is a constant specific to the family of basis set used. We adopted the value of *α* for ground-state Ahlrichs-basis set extrapolations, namely *α* = 10.39 for DZ-TZ and *α* = 7.88 for TZ-QZ extrapolations.^187^

The electron-correlation contribution *E*_C_ was obtained from the differences between CCS and SCS-CC2 or CCSDR(3) total excited-state energies. Those contributions were extrapolated to the CBS limit with the familiar formula for the correlation energy (*E*_C_):^188^3
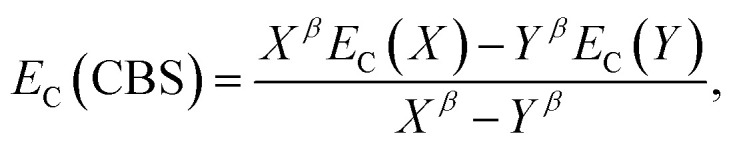
where the basis-set specific constant *β* has values of 2.40 (DZ-TZ) or 2.97 (TZ-QZ), respectively.^187^

**Fig. 4:**
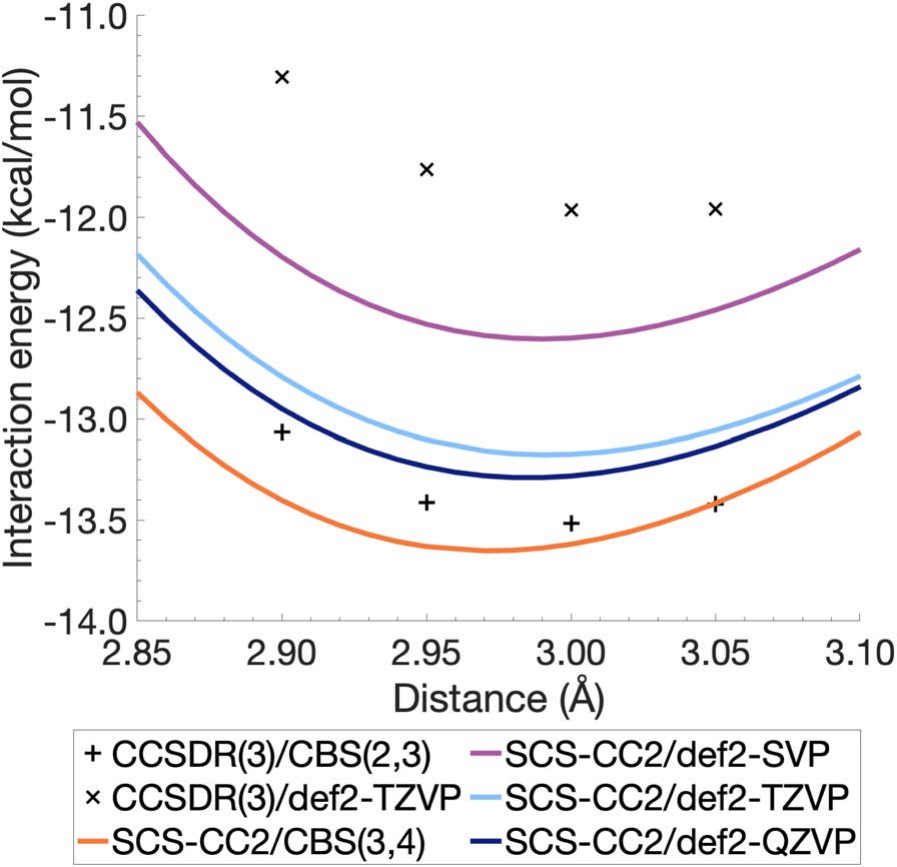
Benzene excimer interaction energies around the minimum energy well for SCS-CC2 and CCSDR(3), including CBS-extrapolated results.

The resulting energies *E*_CCS_(CBS) and *E*_C_(CBS) were added together to obtain the excited-state total energies. These were then used to calculate the interaction energies at the CBS level. Ideally, the basis sets used in such extrapolations should be as large as technically possible. For CCSDR(3), only DZ and TZ calculations were feasible for the benzene dimer, while TZ and QZ calculations were possible for SCS-CC2. The resulting CCSDR(3)/CBS(2,3) interaction energies for the same four select intermolecular distances discussed for CCSDR(3)/def2-TZVP in the previous section are shown in Fig. 4 (numerical values in Table S2†) alongside the SCS-CC2 curves with truncated Ahlrichs basis sets and CBS(3,4). SCS-CC2/CBS interaction energies are slightly more negative than CCSDR(3)/CBS for three of the four points with the differences ranging from 0.34 kcal mol^−1^ (*r* = 2.90 Å) to 0.1 kcal mol^−1^ (*r* = 3.00 Å). Both levels of theories agree for *r* = 3.05 Å with Δ*E* = −13.42 kcal mol^−1^. The agreement between both levels is therefore better near the minimum region of CCSDR(3)/CBS, which lies close to 3 Å (see Fig. 4).

Fig. 4 also shows parts of the dissociation curves of the three truncated basis set levels for SCS-CC2. We can clearly rule out the DZ level from further consideration. The TZ and QZ curves are relatively close to one another with the difference between both their minima being only 0.11 kcal mol^−1^, suggesting the result is close to convergence (Table S1†). The SCS-CC2/def2-TZVP minimum lies 0.34 kcal mol^−1^ above the CCSDR(3)/CBS interaction energy at *r* = 3.00 Å. In contrast, the SCS-CC2/def2-QZVP minimum is only 0.23 kcal mol^−1^ higher. Considering that the CCSDR(3)/CBS interaction energy at *r* = 3.00 Å is −13.52 kcal mol^−1^, the percentage errors for the SCS-CC2/TZ, QZ and CBS(3,4) minima are only 2.5, 1.7 and 0.96%, respectively; see Table 2 for the SCS-CC2/CBS(3,4) minimum.

**Table 2:** SCS-CC2 values for truncated basis sets and at the CBS limit for all excimer structures. The CBS values serve as reference values for the subsequent TD-DFT benchmark study

Basis	Benzene	Naphthalene	Anthracene	Pyrene
**Dissociation energy *D*_e_ (kcal mol^−^** ^**1**^ **)**				
def2-TZVP	13.18	29.18	29.86	35.64
def2-QZVP	13.29	28.50	28.84	34.12
CBS(3,4)	13.65	28.32	28.40	33.29

**Equilibrium distance *r*_e_ (Å)**				
def2-TZVP	2.99	3.06	3.20	3.20
def2-QZVP	2.99	3.05	3.19	3.20
CBS(3,4)	2.97	3.04	3.18	3.19

When choosing a reliable benchmark level of theory for the subsequent TD-DFT study, the accuracy of a method must be considered alongside the time and resources needed to treat the system sizes involved in this study as well as the large number of points needed to generate smooth dissociation curves. As SCS-CC2/def2-QZVP calculations are feasible across all excimer systems, obtaining SCS-CC2/CBS(3,4) curves offers the potential for a reference at a standard comparable with ground-state studies. Ideally, to establish the accuracy of using this level of theory, we would want to obtain CCSDR(3)/def2-TZVP data for the larger excimer systems to offer a comparison. However, at this stage, our computational resources prevent this proposed extension. Given the low percentage errors for the benzene test case for truncated basis sets, discussed above, we obtain SCS-CC2 values for all excimers with TZ, QZ and CBS(3,4). Table 2 lists the values of *D*_e_ and *r*_e_ from minima across these basis set treatments, while the corresponding dissociation curves are given as Fig. S2–S5.† Without higher-level data for all systems we cannot make a definitive statement as to the accuracy of these results. That being said, prior to extrapolation *r*_e_ appears already close to convergence across all systems and *D*_e_ differs within 0.11 to 1.52 kcal mol^−1^.

Our main incentive for the TD-DFT benchmark is to assess the performance of modern TD-DFT methods, with and without applied dispersion corrections, based on their resulting dissociation energy curves. For this purpose, we choose SCS-CC2/CBS(3,4) as the reference level for the subsequent study. We would like to point out that the curves discussed herein are an improvement on what has been acceptable in the field as binding energies with basis sets of QZ quality or higher being rare for aromatic exciplexes. Nevertheless, we recommend a future study dedicated solely to (single-reference) WFT methods that addresses CBS extrapolations and BSSE corrections more in detail. As such studies are beyond the scope of this work, we continue with the discussion of the TD-DFT results.

## 4 Benchmarking TD-DFT methods

Having established SCS-CC2/CBS(3,4) as a reference method for the four excimer models, it is now possible to move on to benchmarking various DFAs. Of the four excimers (Fig. 2), benzene is the smallest and has therefore received the most attention in previous computational studies.^5,90,116,120,121,133,189^ While the other excimers have received some attention, they are typically studied individually^92,93,104,118,119^ rather than comparatively.^7,8,90^ The binding of an excimer involves a change in the geometry upon excitation with the eclipsed dimer, also called the “perfect sandwich” structure,^3,190^ widely accepted as the most stable conformation of an excimer.^7,8,88,133,190^ The excitation responsible for excimer formation causes a displaced ground-state dimer to move into this eclipsed form, which is also associated with a reduction in distance between the monomers.^3,5^ The most energetically stable intermolecular separation as determined experimentally for aromatic excimers is reported in the range of 3.0–3.6 Å.^4^ Our SCS-CC2/CBS(3,4) *r*_e_ values, reported in Table 2, fall within 0.03 Å of this range which is reasonable given high-accuracy theoretical calculations tend to predict shorter *r*_e_.^7^

The close and parallel stacking of the eclipsed formation facilitates excimer binding interactions: electrostatics, Pauli-repulsion, CT, exciton coupling and London dispersion.^8^ For example, parallel stacking and short intermolecular distance increases orbital overlap, promoting exciton delocalisation.^102^ The interplay of these various interactions may present a challenge for DFT methods to properly describe the dissociation energies and inter-molecular distances. The main focus of this section is to investigate DFAs with this in mind. Before we discuss each model system individually, we study the impact of the amount of FE on the TD-DFT dissociation energies. Each model system is then first discussed without the addition of dispersion corrections before the impact of said corrections is studied separately. This section ends with an overarching discussion across all four dimers by means of statistical analysis.

### 4.1 Dependence on Fock exchange

**Fig. 5:**
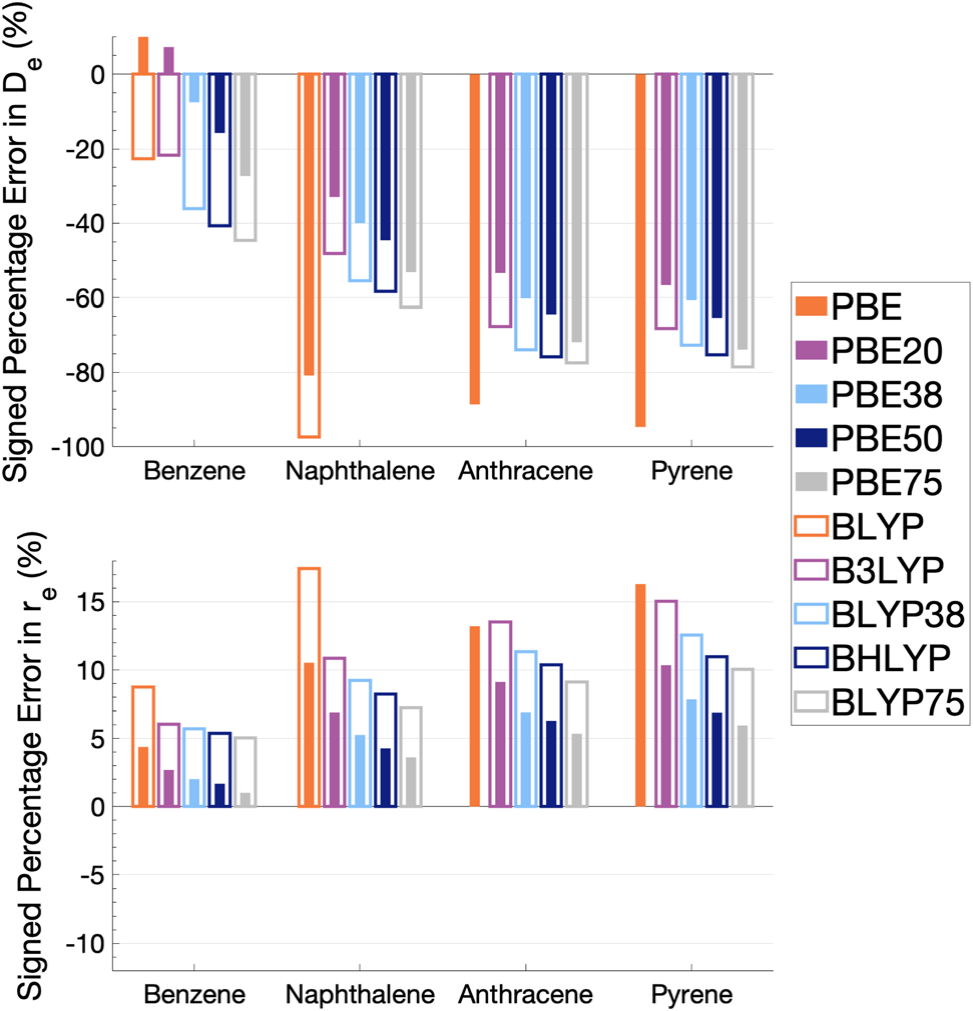
Percentage error in dissociation energy (*D*_e_, top) and equilibrium inter-monomer separation (*r*_e_, bottom) of each excimer with respect to SCS-CC2/CBS(3,4) for varied Fock exchange with PBE and BLYP exchange-correlation functionals. BLYP results are not shown for anthracene and pyrene excimers as they gave repulsive dissociation curves.

In this section, we analyse the influence of FE on the description of excimer binding by global hybrids. This analysis was performed by varying percentages of FE, between 0 and 75%, for two underlying exchange-correlation (XC) approximations, PBE and BLYP. The resulting functionals of varied FE are detailed in Table 1. PBE and BLYP were chosen as they are the XC approximations behind many popular (double) hybrids, and as using two different approximations may help us to better indicate the individual influence of FE or the underlying XC expression. The functionals are analysed through their ability to recover the minimum of the dissociation curve of each excimer which can be separated into the equilibrium distance (*r*_e_) between the two monomers and the stability of the dimers represented by the dissociation energy (*D*_e_). The ability of each method to describe these parameters is assessed by quantitative comparison to those calculated with our SCS-CC2/CBS(3,4) reference by use of signed percentage error:4
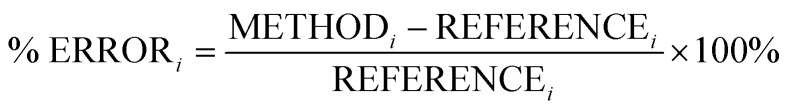
where *i* represents either *D*_e_ or *r*_e_. In this definition of percentage error, positive values represent overestimation while negative ones represent underestimation of the quantity. Fig. 5 details the resulting signed percentage errors for *D*_e_ (top panel) and *r*_e_ (bottom panel). The numerical values and corresponding dissociation energy curves are given in Section S1.4 in the ESI.†

As Fig. 5 shows, the percentage errors in *D*_e_ and *r*_e_ are considerably large across all systems regardless of FE percentage. Almost all functionals underestimate *D*_e_, with a magnitude that tends to increase with amount of FE and system size, averaging across structures these errors range from about −66 to −34%. The size-dependence of the error can be partially explained with the fact that dispersion effects are expected to increase with system size^191^ and that dispersion corrections have not been applied at this stage. Percentage errors in *r*_e_ are universally positive with magnitudes that decrease with percentage of FE, with errors averaged across structures ranging from 4 to 13%. Overall, with an increase in FE, errors in *D*_e_ and *r*_e_ increase and decrease, respectively. Despite the overall poor performance from global hybrids regardless of FE percentage we can still gain an insight into the impact FE has for the complicated effects of excimer binding.

Without FE, generalised-gradient approximation (GGA) errors are larger than those of global hybrids (−97.4 to 12.6% error range in *D*_e_) with BLYP unable to bind the anthracene and pyrene excimers. As GGAs are well-known to produce large errors for excited states and offer no exception here, they are not explored in the subsequent benchmarking study.

Functionals with large amounts of FE tend to worsen the description of excimer binding across the four tested systems. Functionals with 20% FE yield the smallest absolute errors in *D*_e_ for each excimer, but the errors do increase with system size, yielding errors that range from −68.2 to 7.3%. Functionals containing 75% FE (−78.6 to −27.3% error range in *D*_e_) give the largest absolute errors of the global hybrids. An increase in *D*_e_ error with FE is somewhat surprising given that global hybrids with large amounts of FE generally describe CT and other long-range excitation effects better than smaller amounts of FE.^60^ High percentages of FE do improve the description of *r*_e_ which may reflect the improvement of these long-range excitation effects. However this improved description of *r*_e_ cannot overcome the poor description of *D*_e_ by these functionals and an overall error that increases with system size. Due to the opposing effects of FE on the recovery of both minimum characteristics, the most balanced functionals are those with 37.5% FE.

The PBE-based functionals yield larger dissociation energies than BLYP-based functionals which, as most of the functionals underestimate the excimer stability, more closely resemble the reference well; for example, PBE20 yields errors in *D*_e_ ranging between −56.6 and 7.3% while B3LYP errors range between −68.2 and −21.7%. This difference in behaviours is also exhibited by GGAs, where PBE binds all four excimers whereas BLYP is unable to bind the anthracene and pyrene excimers. Interaction energy curves of CCS, which can be thought of as the HF equivalent for electronic excited states, were plotted to offer a comparison to a method with no electron-correlation but 100% FE (see Fig. S7†). The CCS/def2-TZVP interaction energy curves possess only very shallow minima for the anthracene (*D*_e_ = 0.31 kcal mol^−1^) and pyrene (*D*_e_ = 0.70 kcal mol^−1^) excimers indicating that electron correlation plays a significant role in the stabilisation of those systems. PBE-based functionals can be thought to better describe the electron correlation contributions to the excimer states than BLYP based functionals. The difference between the two underlying XC functionals for the excimer state therefore parallels that of ground states, where PBE is more attractive than BLYP in the treatment of NCIs.^42,84,192^

Despite the generally improved performance of PBE-based functionals, DFAs based on both XC expressions greatly underestimate the dissociation energy and overestimate *r*_e_ with error trends that increase with FE and system size, indicating the requirement of more sophisticated functionals for an accurate description of excimer binding. With increasing FE, errors in *D*_e_ increase while errors in *r*_e_ decrease such that the ability of a global hybrid TD-DFA to describe each quantity seems to require a trade-off in accuracy to the other. As both *D*_e_ and *r*_e_ must be correctly described in order to predict excimer binding, global-hybrid TD-DFAs do not offer reliable results. Methods from the higher rungs of Jacob's Ladder and additive dispersion corrections have been effective in addressing some of short-comings associated with global hybrids for ground states, so their performance will be addressed in the following sections.

### 4.2 Individual discussion of excimer models

**Fig. 6:**
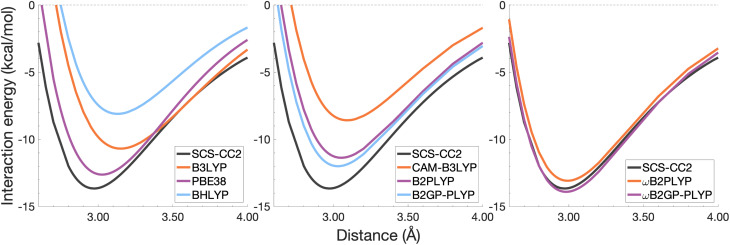
Dissociation energy curves of the lowest-lying singlet excited state of the fully-stacked benzene dimer.

**Fig. 7:**
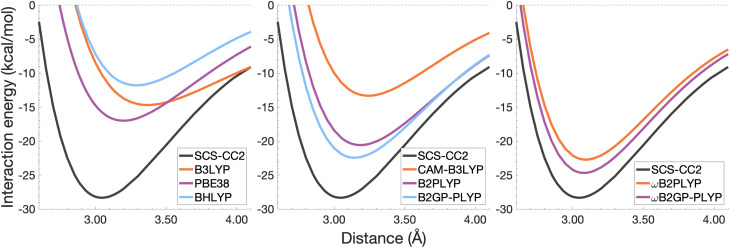
Dissociation energy curves of the lowest-lying singlet excited state of the fully-stacked naphthalene dimer.

**Fig. 8:**
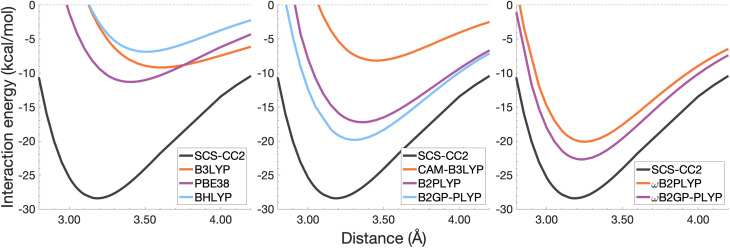
Dissociation energy curves of the lowest-lying singlet excited state of the fully-stacked anthracene dimer.

**Fig. 9:**
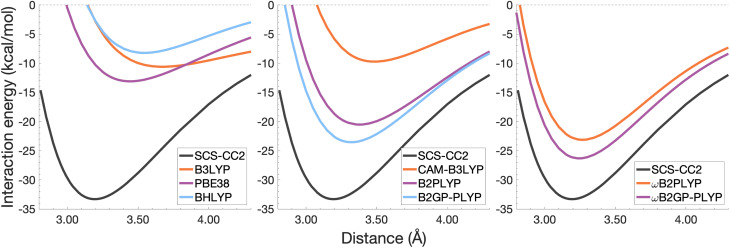
Dissociation energy curves of the lowest-lying singlet excited state of the fully-stacked pyrene dimer.

In this section, the interaction energy curves for each excimer across a range of TD-DFAs are analysed relative to the reference of SCS-CC2/CBS(3,4). The chosen range of TD-DFAs is based on either popularity, established accuracy for single-molecule excitations, or novelty; they are: B3LYP, PBE38, BHLYP (global hybrids), CAM-B3LYP, ωB97X (RS hybrids), B2PLYP, B2GP-PLYP (global DHDFAs), ωB2PLYP and ωB2GP-PLYP (RS-DHDFAs). These functionals occupy the top two rungs of Jacob's Ladder with varying exchange-correlation components and FE percentages, with and without RS, as detailed in Table 1. For all the dissociation curves discussed, the values associated with the minima, *i.e. D*_e_ and *r*_e_, are listed in Table 3. Dissociation curves are shown in Fig. 6–9. ωB97X interaction energy curves are not shown here, but instead in Fig. S6† due to observed problems with getting smooth curves that could not be fixed with any of the usual convergence techniques. ωB97X will however be discussed as part of the overall statistical analysis (Section 4.4) with the values based on the actually observed minima under the assumption that they are good approximations to the true minima. Hereafter, deviations from the reference and associated percentage errors will be given as absolute values. Indication of under- or overestimation will be discussed qualitatively.

**Table 3:** Dissociation energies (*D*_e_ in kcal mol^−1^) and equilibrium inter-monomer distances (*r*_e_ in Å) for each excimer across the nine uncorrected functionals. SCS-CC2/CBS(3,4) numbers are shown as a reference

Method	Benzene		Naphthalene		Anthracene		Pyrene	
	*D*_e_	*r*_e_	*D*_e_	*r*_e_	*D*_e_	*r*_e_	*D*_e_	*r*_e_
Reference	13.65	2.97	28.32	3.04	28.40	3.18	33.29	3.19
B3LYP	10.68	3.15	14.69	3.37	9.19	3.61	10.60	3.67
PBE38	12.62	3.03	17.00	3.20	11.31	3.40	13.09	3.44
BHLYP	8.10	3.13	11.79	3.29	6.88	3.51	8.22	3.54
CAM-B3LYP	8.58	3.09	13.32	3.24	8.18	3.45	9.71	3.50
ωB97X^a^	11.99	3.08	18.95	3.24	16.01	3.36	17.97	3.37
B2PLYP	11.36	3.05	20.58	3.19	17.23	3.36	20.51	3.38
B2GP-PLYP	11.99	3.03	22.43	3.14	19.82	3.31	23.53	3.32
ωB2PLYP	13.07	2.99	22.72	3.09	20.10	3.25	23.11	3.27
ωB2GP-PLYP	13.90	2.98	24.67	3.07	22.70	3.23	26.29	3.25

a Approximated and based on observed minima; see explanation in text and problematic curves in Fig. S6.

#### 4.2.1 The benzene excimer

For the benzene excimer, excited-state interaction energies calculated across varied inter-monomer distances at the SCS-CC2/CBS(3,4) level of theory (Fig. 6) yield a dissociation energy of 13.65 kcal mol^−1^ at 2.97 Å (*r*_e_). The ability to describe the shape and depth of the potential energy well differs from DFA to DFA. The assessed global hybrids (left panel in Fig. 6) consistently underbind the benzene excimer, underestimating *D*_e_ by 1.03–7.96 kcal mol^−1^ (8–58% error) and overestimating *r*_e_ by 0.06–0.20 Å (2–7% error), some of the largest errors for this system. PBE38 is somewhat of an exception to the other global hybrids with a well-depth (12.62 kcal mol^−1^) and position (3.03 Å) in line with higher-rung double hybrids. However, as discussed in the FE study, global hybrids are not capable of reliably capturing the challenging interactions in excimer binding. Poor performance of global hybrids is consistent with their inability to accurately describe the CT, exciton coupling and dispersion interactions inherent to excimer binding.^8,42,79,193^ As we will see in the following sections, the performance of PBE38 is not as favourable for the larger systems tested.

The long-range correction in CAM-B3LYP reduces the overestimation in *r*_e_ compared to its uncorrected counterpart B3LYP with an improvement from a deviation of 0.18 to 0.12 Å (central panel in Fig. 6); however it also worsens the description of *D*_e_ (2.97 kcal mol^−1^ underestimation for B3LYP *vs.* 5.08 kcal mol^−1^ for CAM-B3LYP). Double hybrids yield an improved description of interaction energies along the dissociation curve, with B2GP-PLYP being closer to the reference than B2PLYP (central panel in Fig. 6), both of which underestimate *D*_e_ by 1.66 and 2.29 kcal mol^−1^, respectively (12 and 17% error).

Double hybrids provide a more balanced description of the excimer binding than global and RS hybrids. This improvement is consistent with benchmarking trends of double-hybrid robustness for excitation energies and absorption spectra due to their perturbative correction.^47,66,77^ Improved performance by DHDFAs additionally parallels the finding that only double hybrids were able to properly describe an exciton-coupled ECD spectrum,^79^ for which WFT methods had to be applied earlier.^194^ However, as recently re-emphasised, global double hybrids still fail to correctly describe CT excitations.^63^ The combination of range-separation and perturbative correction improves the description of the benzene excimer dissociation energy curve, giving the curves closest to the reference at all intermolecular distances (right panel in Fig. 6). The assessed RS-DHDFAs, ωB2PLYP and ωB2GP-PLYP, improve upon their uncorrected counterparts, with the former underestimating *D*_e_ by 0.58 kcal mol^−1^ (4% error) and the latter slightly overestimating it by 0.25 kcal mol^−1^ (2% error). Close comparison of RS-DHDFAs with the reference interaction energy curves of the benzene excimer display potential to corroborate claims of their robustness for local-valence and long-range excitations.^52,53,62,64^ In summary, it is observed that, for the benzene excimer, the description of binding is improved by climbing Jacob's Ladder with rung-five functionals showing considerable improvement over those belonging to rung four (see Table 1 for the Jacob's Ladder classification of each assessed functional).

#### 4.2.2 The naphthalene excimer

The naphthalene excimer is more than twice as stable as the benzene excimer, yielding a SCS-CC2/CBS(3,4) dissociation energy of 28.32 kcal mol^−1^ at 3.04 Å (Fig. 7). When assessing DFA dissociation energies, one has to consider the well-documented issue of conventional TD-DFT methods often struggling to correctly order the first two excited states of the naphthalene monomer.^195^ For instance, TD-B3LYP and TD-BHLYP incorrectly predict the ^1^L_a_ state of the monomer to be lower than the ^1^L_b_ state.^52,58,195^ In the fully-stacked dimer, the first excimer state has, in fact, ^1^L_a_ character, but at the dissociation limit this turns out to be the second excited state.^7,118^ Indeed, we observe this for our reference method and most other methods, but want to point out that the aforementioned problem for the naphthalene monomer is also observed here, which can lead to the incorrect calculation of dissociation energies for B3LYP and BHLYP if this problem is not spotted. This is particularly a problem when a calculation is carried out without any symmetry, but less so if the programs used distinguish between excited-state symmetries, as the symmetries of the first two excited states differ.

The chosen exchange-correlation functionals uniformly predict a weaker binding with absolute errors of *D*_e_ ranging between 3.65 and 16.53 kcal mol^−1^ (13–58% error). *r*_e_ is largely overestimated with absolute deviations ranging from 0.03 to 0.33 Å (0.3–11% error). The fourth-rung functionals behave differently between naphthalene and benzene excimers whereas fifth-rung functionals exhibit similar performance trends. For the naphthalene excimer PBE38 offers an *r*_e_ closer to the reference (0.16 Å deviation, 5% error) than the BLYP based functionals, however underestimation of *D*_e_ is on par with other DFAs belonging to this rung (40% error), consistent with results from our previous FE study in Section 4.1. B3LYP binds the excimer with a *D*_e_ comparable to those of other global hybrids (48% error) while its long-range corrected counterpart, CAM-B3LYP, again improves the description of *r*_e_ (reduced deviation from 0.33 to 0.20 Å) but yields *D*_e_ akin to the global hybrids (53% error). While the relative trends between rung-four functionals differ between benzene and naphthalene excimers, the finding remains that global hybrids provide the worst results.

Double hybrids improve the description of excimer binding considerably with absolute errors in the order of 21–27% and 3–5% for *D*_e_ and *r*_e_, respectively. Further improvement on conventional DHDFAs results from the inclusion of range-separation with absolute errors in the order of 13–20% and 1–2% for *D*_e_ and *r*_e_, respectively. Despite the closer resemblance of RS-DHDFAs curves with the reference curve (right panel in Fig. 7) the binding strength is insufficiently described. The best method, ωB2GP-PLYP underestimates the dissociation energy by 3.65 kcal mol^−1^, which is a larger error than for the benzene dimer in Section 4.2.1. Most likely, this error can be attributed to missing dispersion, as its importance increases with the number of electrons.^191^ This will be further discussed in Section 4.4.

#### 4.2.3 The anthracene excimer

The anthracene excimer is slightly more stable than the naphthalene excimer, with an SCS-CC2/CBS(3,4) dissociation energy of 28.40 kcal mol^−1^ at a larger equilibrium monomer separation of 3.18 Å (Fig. 8). The herein tested TD-DFAs uniformly underbind the anthracene excimer with absolute errors in *D*_e_ between 5.71 and 21.52 kcal mol^−1^ and overestimate *r*_e_ by between 0.05 and 0.43 Å. Trends in functional performance are similar to those of the naphthalene excimer, although with larger errors in *D*_e_. Each functional predicts a smaller excimer dissociation energy for the anthracene excimer than it does for the naphthalene excimer, so comparison with the larger reference value for the anthracene excimer results in larger errors for *D*_e_ (20–76%).

It was previously noted that the *D*_e_ of the anthracene excimer being larger than that of the naphthalene excimer may be due to a larger dispersion contribution to the excimer binding.^7^ Given that the TD-DFT methods discussed in this sections are unable to describe dispersion, this further supports the study of dispersion-corrected functionals for excimer binding in Section 4.3.

#### 4.2.4 The pyrene excimer

The pyrene excimer (Fig. 9) is more stable than the anthracene excimer, with an SCS-CC2/CBS(3,4) dissociation energy of 33.29 kcal mol^−1^ at 3.19 Å. Similarly to the naphthalene dimer, we also observed the wrong order of states at the dissociation limit for some functionals, something that can be avoided when using symmetry in the calculation.

Each functional predicts similar dissociation energies as for the anthracene case (within 1.33–3.71 kcal mol^−1^) which, relative to the difference between the reference *D*_e_ values (4.89 kcal mol^−1^), yields remarkably similar errors (21–75%). The trends observed for the naphthalene and anthracene excimers hold true for the pyrene excimer, with an improved description of excimer stabilisation moving up Jacob's Ladder.

The analysis of *r*_e_ is similar between pyrene and anthracene, showing errors for the same methods within 0.05 Å. Range-separation improves the equilibrium distance, with RS-DHDFAs predicting *r*_e_ within absolute error of 0.08 Å (3% error).

To summarise Section 4.2, the relative performance of the tested functionals follows a general trend: global hybrids and CAM-B3LYP perform the worst in terms of *D*_e_ and *r*_e_, with a slight improvement in *r*_e_ through range-separation; double hybrids improve upon the description of both well characteristics while range-separated double hybrids show the best description of both characteristics across the tested functionals. We saw an increase in the error of *D*_e_ with system size which may be due to the lack of properly treating dispersion interactions. In the following section we explore the application of ground-state optimised dispersion corrections to assess their potential in accounting for the missing dispersion in excimer binding.

### 4.3 Impact of dispersion corrections

So far we have only discussed dispersion-uncorrected results. As dispersion-uncorrected TD-DFAs are able to partially predict excimer binding it is clear that interactions beyond dispersion are important, which aligns with the findings from an energy decomposition analysis reported in 2018.^8^ In our introduction we have pointed out how some studies make use of dispersion-uncorrected methods for the treatment of excimers, but our results clearly show that dispersion effects should not be neglected. Herein, we demonstrate the impact of ground-state dispersion corrections for the two extreme cases in our study, namely the benzene dimer as the smallest and the pyrene dimer as the largest system.

Ground-state dispersion corrections are often applied to excited-state studies justified by seemingly good outcomes from a few studies.^5,114,115^ However, it is reasonable to expect dispersion interactions to change upon electronic excitation^113^ rendering any benefit application-dependent. Herein, we test these justifications for two schemes of additive DFT-D type dispersion corrections as applied to the excimer state without any state-specific adjustment. The DFT-D3(BJ) and DFT-D4—hereafter dubbed “D3(BJ)” and “D4”—dispersion energies were calculated for all points along the dissociation curves and added to the respective total excited-state energies. The analysis of these dispersion-corrected dissociation energy curves focuses on four functionals B3LYP, CAM-B3LYP and B2PLYP and ωB2PLYP, *i.e.* two global functionals and their range-separated counterparts (minima given in Table 4). A more generalised picture across all eight dispersion-corrected functionals will be provided in the overall statistical analysis in Section 4.4.

**Table 4:** Dissociation energies (*D*_e_ in kcal mol^−1^) and equilibrium inter-monomer distances (*r*_e_ in Å) for the benzene and pyrene excimers for select dispersion-corrected and uncorrected functionals. SCS-CC2/CBS(3,4) numbers are shown as a reference

Functional	Uncorrected		DFT-D3(BJ)		DFT-D4	
	*D*_e_	*r*_e_	*D*_e_	*r*_e_	*D*_e_	*r*_e_
**Benzene excimer**						
Reference	13.65	2.97	—	—	—	—
B3LYP	10.68	3.15	21.24	3.03	21.47	3.03
CAM-B3LYP	8.58	3.09	15.24	3.04	15.77	3.03
B2PLYP	11.36	3.05	16.94	3.00	16.96	3.01
ωB2PLYP	13.07	2.99	13.20	2.99	13.19	2.99

**Pyrene excimer**						
Reference	33.29	3.19	—	—	—	—
B3LYP	10.60	3.67	33.41	3.31	34.37	3.31
CAM-B3LYP	9.71	3.50	27.39	3.33	28.41	3.32
B2PLYP	20.51	3.38	34.28	3.26	34.60	3.27
ωB2PLYP	23.11	3.27	23.63	3.27	23.66	3.27

#### 4.3.1 Binding region of potential energy well

**Fig. 10:**
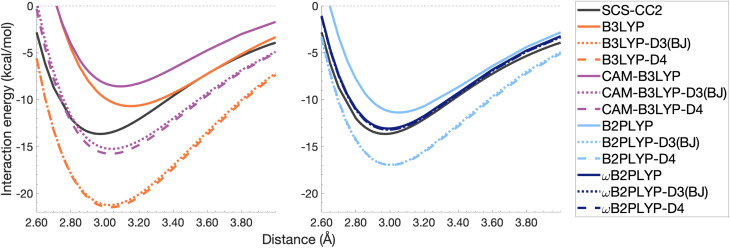
Dissociation energy curves of the lowest-lying singlet excited state of the fully-stacked benzene dimer for selected functionals with and without dispersion corrections.

For the benzene excimer, both dispersion corrections overcorrect the binding predicted by each TD-DFA except ωB2PLYP (Fig. 10). Whether D4 or D3(BJ) is closer to the reference depends on whether the dispersion-corrected method under- or overbinds the excimer; if the former is the case, the D4 version is closer to the reference, but in the latter case the D3(BJ) variant is closer. Across the four functionals shown here, absolute deviations in *D*_e_ range from 0.46 to 7.82 kcal mol^−1^ for D4 and from 0.45 to 7.59 kcal mol^−1^ for D3(BJ). The corrections offer an improved but overestimated description of the optimal inter-monomer separation for B3LYP, CAM-B3LYP, and B2PLYP with errors between 0.03 and 0.07 Å for D3(BJ) and between 0.04 and 0.06 Å for D4. However, the improvement to *r*_e_ is drastically over-shadowed by the greatly overstabilised dissociation energy. Dispersion-corrected and -uncorrected ωB2PLYP give the same overestimation of *r*_e_ by 0.02 Å. As the smallest of the model systems, benzene excimer binding contains a smaller dispersion contribution than the larger models. With less dispersion to account for, more complex functionals are able to reasonably account for the other contributions to the dissociation energy without dispersion correction. The ground-state optimised corrections, thus, have a tendency to overestimate the dispersion contribution to the benzene excimer dissociation energy.

**Fig. 11:**
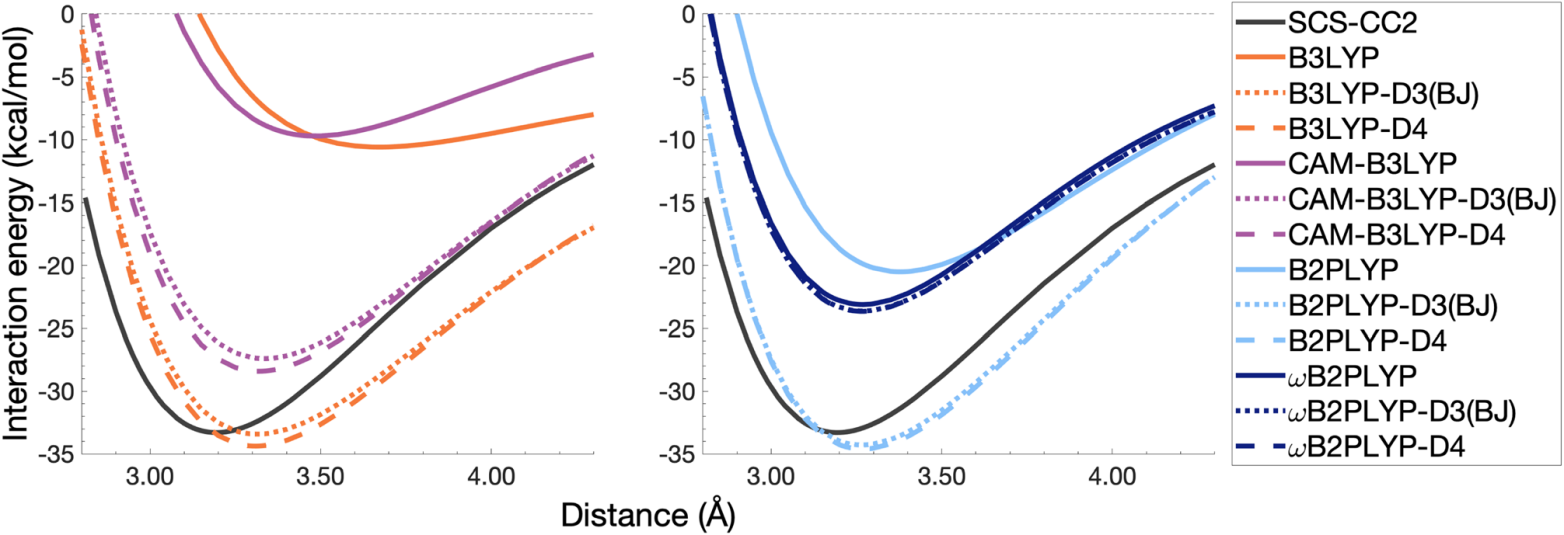
Dissociation energy curves of the lowest-lying singlet excited state of the fully-stacked pyrene dimer for selected functionals with and without dispersion corrections.

The pyrene excimer is larger and more strongly bound than the benzene excimer, which the selected uncorrected TD-DFAs fail to predict, underestimating *D*_e_ in the range of 10.18–23.58 kcal mol^−1^ (Fig. 11). For this larger system, dispersion-corrected TD-DFAs offer a more balanced improvement to the description of the pyrene excimer than for the benzene excimer: D4 and D3(BJ) corrected functionals over and underestimate *D*_e_ with absolute errors ranging from 1.08 to 9.63 kcal mol^−1^ and from 0.12 to 9.66 kcal mol^−1^, respectively. D4 corrections predict stronger binding than D3(BJ) for all functionals, so as with benzene, the preference of D3(BJ) or D4 depends on whether the dispersion-corrected TD-DFA over or underbinds the pyrene excimer.

Both dispersion corrections considerably improve the B3LYP dissociation energy to within 1.08 kcal mol^−1^ accompanied by an improved *r*_e_ with an overestimation of 0.12 Å. Dispersion-corrected CAM-B3LYP does not show the same improvement to the dissociation energy as its global counterpart, yielding an underestimation of up to 5.90 kcal mol^−1^ for *D*_e_ and an overestimation of *r*_e_ by 0.14 and 0.13 Å for D3(BJ) and D4, respectively. B2PLYP-D3(BJ) and -D4 give dissociation energies with errors comparable to dispersion-corrected B3LYP (overbound by 0.99 and 1.31 kcal mol^−1^), but with a smaller geometry error (overestimation of 0.07 and 0.08 Å). Dispersion corrected ωB2PLYP offers only a small improvement to the uncorrected functional such that ωB2PLYP-D3(BJ) and ωB2PLYP-D4 underbind the pyrene excimer by 9.66 and 9.63 kcal mol^−1^, respectively, and overestimate *r*_e_ by 0.08 Å. A negligible impact of D3(BJ) and D4 on both ωB2PLYP and ωB2GP-PLYP has also been noticed in ground-state benchmarking on the complete GMTKN55 (ref. 27) database and its NCI category.^152^

For the larger system size of the pyrene excimer, the ground-state dispersion corrections considerably improve TD-DFA dissociation energies and equilibrium distances. This is likely why others^5,93,117,119^ have recommended the use of ground-state optimised dispersion corrections for the calculation of this excimer's dissociation energy. While DFT-D type dispersion corrections offer some improvement to the description of excimer binding, their performance is far from a black-box solution for excited states. Inconsistent improvement by ground-state dispersion corrections for excited states reinforces the need for state-specific dispersion corrections for reliable and predictive TD-DFT methods.

#### 4.3.2 Unphysical repulsion in the mid-range

**Fig. 12:**
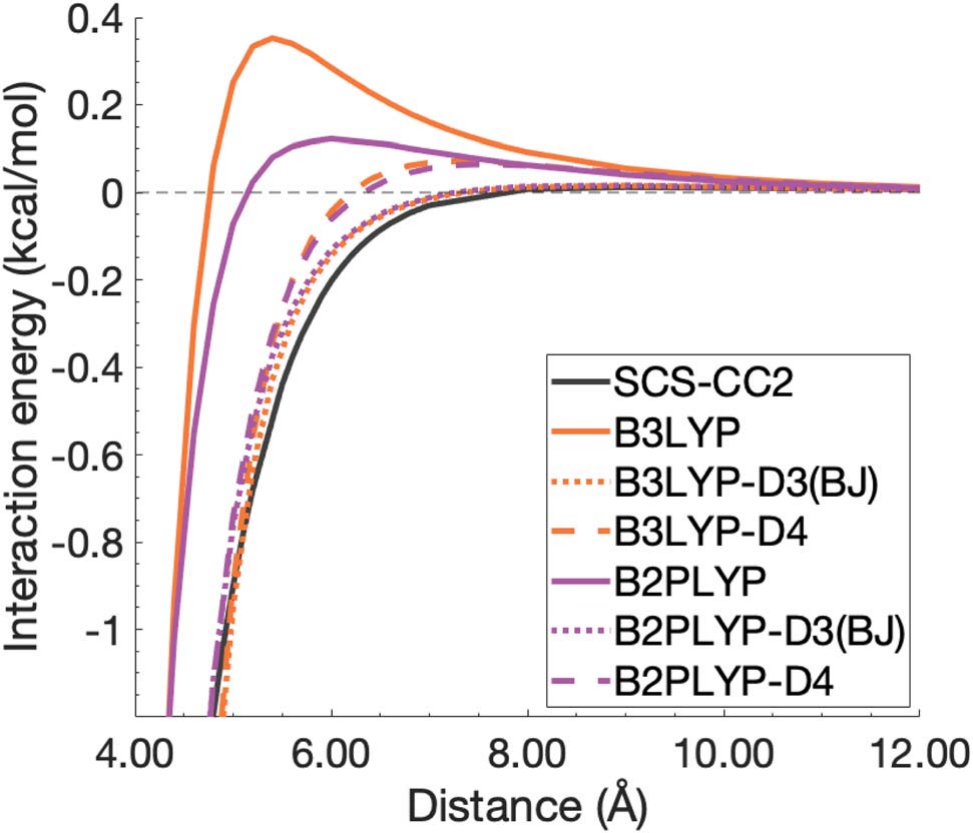
Unphysical repulsive region for the benzene excimer state dissociation curve and its correction by ground-state optimised dispersion corrections.

Beyond the binding region, in the medium-to-long range inter-monomer separation, TD-DFA interaction energy curves falsely predict a repulsive region. For the benzene excimer, all functionals as well as SCS-CC2/def2-TZVP exhibit that positive region falling between 4.3 Å and the asymptote. An extrapolation of SCS-CC2 to the CBS limit [CBS(3,4)] mostly corrects the unphysical repulsion of SCS-CC2/def2-TZVP, reducing the repulsion to less than 0.01 kcal mol^−1^, which is way within the expected numerical noise for that method and a negligible value (see Fig. S13†). Naphthalene and anthracene excimers exhibit a similar repulsive region for global hybrids and range-separated hybrids (see Fig. S14–S17† for further details). Herein, we focus on the benzene excimer by observing B3LYP and B2PLYP with and without dispersion corrections in the range between 4.00 and 12.00 Å (Fig. 12). The extent of the repulsion for dispersion-uncorrected B3LYP is over twice that of B2PLYP, with maxima of 0.35 and 0.12 kcal mol^−1^, respectively. The D3(BJ) correction reduces the repulsion yielding maxima of up to 0.02 kcal mol^−1^, which brings the two functionals into close comparison with each other (maxima within 0.001 kcal mol^−1^ of each other). The D4 curves are also comparable in this region, although they exhibit larger maxima than D3(BJ) curves: 0.07 and 0.06 kcal mol^−1^ for B3LYP-D4 and B2PLYP-D4, respectively. The positive behaviour that remains after the applied corrections would not be expected with corrections appropriately parametrised for excited states. Interestingly, in a recent study of exciplex interaction energy curves, a similar unphysical repulsive hump exhibited by ωB97 also saw correction by ωB97X-D3 (ref. 196) with DFT-D3(0), something that has not been noticed by the authors.^102^ As excimer binding is comprised of more than just dispersion effects,^8^ dispersion-uncorrected functionals are able to predict some attraction in the binding region, with more complex functionals performing reasonably in binding the benzene excimer. In the medium-range, however, the dispersion-uncorrected functionals predict an unphysical repulsive region. Medium-range inter-monomer separations appear to be governed by dispersion, and can therefore only be accounted for by inclusion of the dispersion energy missing from the description by dispersion-uncorrected TD-DFAs.

With this closer look at ground-state dispersion corrections for the binding of benzene and pyrene excimers, it is clear that accounting for dispersion is important for accurate calculation of dissociation curves in both the binding region and beyond. While ground-state optimised dispersion corrections offer some improvement to excimer binding, a state-specific reparametrisation for excited states would be necessary for robust and reliable TD-DFA results. An overarching discussion of the performance of dispersion corrections for all benchmarked DFAs is presented in the following section.

### 4.4 Averaged functional performance

**Fig. 13:**
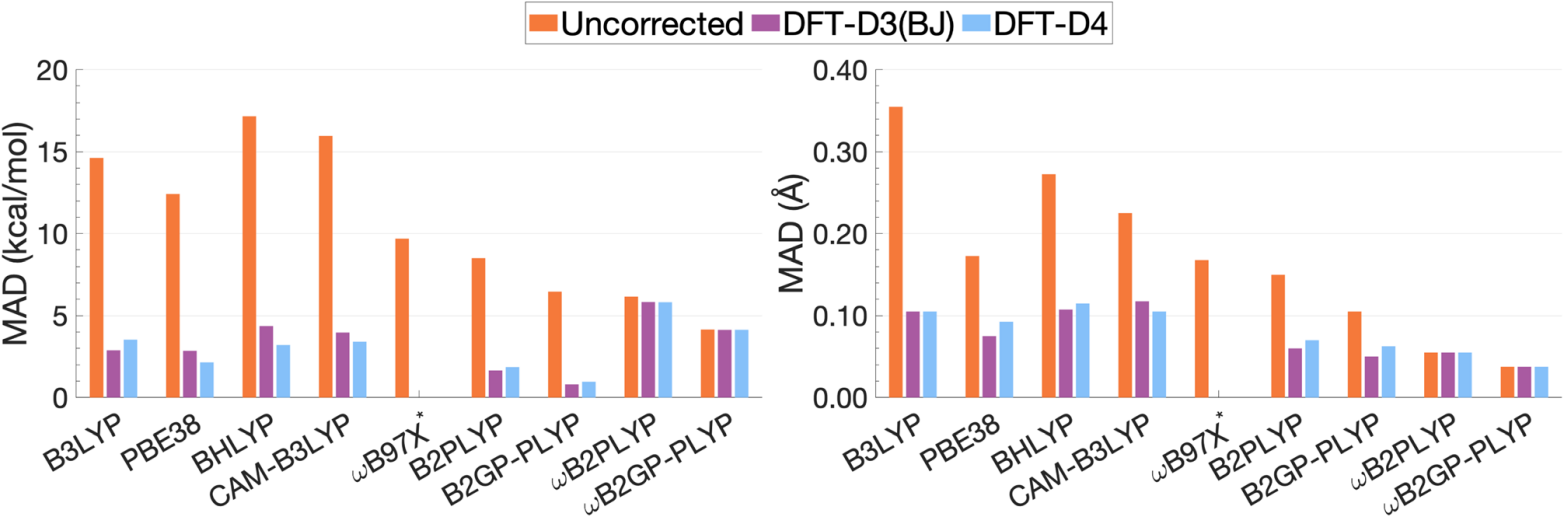
Mean absolute deviations for *D*_e_ (left) and *r*_e_ (right). * We assessed the pure ωB97X. Its various dispersion-corrected variants all depend on slightly different underlying XC expressions,^31,145,196,198–200^ which is why dispersion-corrected results are not provided.

Following our previous analysis of the interaction energy curves, we continue with an overall discussion of each functional's performance averaged across all systems both with and without dispersion corrections. Functional performance is almost universally assessed by mean absolute deviations (MADs) as the metric for benchmarking of quantum-chemical methods.^193^ Here, the MAD for each characteristic averaged for each method is calculated by following the general form:5
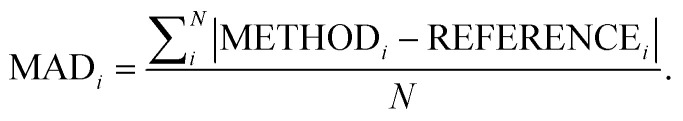
where METHOD_*i*_ and REFERENCE_*i*_ are the values of the property for the *i*th excimer, either *D*_e_ or *r*_e_, and *N* the number of systems. An analysis of MADs is simply presented as a convenient metric to summarise the performance of a method across our four model structures, and we acknowledge a sample size of four does not offer MADs with statistical significance comparable to more comprehensive excited-state benchmarks.^51,55,57,59,163–165,197^ MADs for the nine functionals, with and without D3(BJ) and D4 dispersion corrections, compared to the SCS-CC2/CBS(3,4) reference are presented in Fig. 13, with corresponding numerical values reported in Table S9.†

For the description of *D*_e_ and *r*_e_, dispersion-uncorrected functionals display a descending trend for the MADs while ascending Jacob's Ladder (Fig. 13). It should be noted that, for dispersion-uncorrected functionals, these deviations overwhelmingly correspond to underestimations of *D*_e_ and overestimations of *r*_e_, whereas dispersion-corrected functionals vary in this regard (see ESI†). The largest errors in *D*_e_ correspond to global hybrids and CAM-B3LYP yielding MADs in the order of 14.63 to 17.17 kcal mol^−1^. Similarly, the largest MADs in *r*_e_ range between 0.23 and 0.36 Å and are attributed to CAM-B3LYP, BHLYP and B3LYP, *i.e.* the global and RS hybrids with Becke88 exchange and LYP correlation. PBE38 gives a smaller MAD of 0.17 Å, matching that of the range-separated hybrid ωB97X.

For global hybrids, an increase in FE is generally associated with an increase in MAD for *D*_e_, corroborating the results of the FE study (Section 4.1). This increase in deviation supports the tendency of global hybrids with large amounts of FE to cause issues for describing excitations in TD-DFT.^67–70^ For example, global hybrids with large components of FE tend to produce blue-shifted excitation energies in single molecules.^55,57,66^ Despite the established better description of CT excitations with range-separated hybrids, CAM-B3LYP performs worse than B3LYP. On the other hand, ωB97X, despite the aforementioned problems with obtaining smooth dissociation curves, proves to be the best tested dispersion-uncorrected hybrid in this study with MADs of 9.69 kcal mol^−1^ and 0.17 Å.

We have already mentioned problems in state order for the naphthalene and pyrene dimers in Sections 4.2.2 and 4.2.4, but still need to elaborate more in detail on the fact that the dimers consist of polycyclic aromatic hydrocarbons (PAHs). Single-molecule studies of PAHs have established that the first two π–π* excitations in PAHs—called L_a_ and L_b_ according to Platt^201^—are poorly described by many TD-DFT methods, including global hybrids.^195,202^ RS hybrids can improve the description of L_a_ but blueshift L_b_ excitations.^76,203^ Double hybrids, particularly the latest range-separated ones, have to date yielded the most accurate and balanced description of both states in PAHs.^52,53,58^ This, in addition to the aforementioned better description of exciton coupling,^79^ most likely explains why DHDFAs are the best-performing methods in our study. The global DHDFAs B2PLYP and B2GP-PLYP have MADs of 8.50 and 6.47 kcal mol^−1^ for *D*_e_ and of 0.15 Å and 0.11 Å in *r*_e_. The two dispersion-uncorrected RS-DHDFAs show even better MADs, most likely due to the better description of CT effects, but their MADs in *D*_e_ are still well above the accepted chemical-accuracy threshold of 0.1 kcal mol^−1^ for NCIs with values of 6.16 and 4.15 kcal mol^−1^ for ωB2PLYP and ωB2GP-PLYP. Their values for *r*_e_ are 0.05 and 0.04 Å, respectively.

In conclusion, all tested dispersion-uncorrected functionals fail to recover excimer binding of SCS-CC2/CBS(3,4) quality.

The application of D3(BJ) and D4 dispersion corrections shows significantly reduced MADs across all tested functionals, excluding ωB2(GP-)PLYP, with larger reductions observed for *D*_e_ than for *r*_e_. However, the improvement to excimer binding by the tested dispersion corrections shown through this reduction in MADs is not predictable. While dispersion-uncorrected functionals yield errors due to underestimation of *D*_e_ and overestimation of *r*_e_, dispersion corrections under or overcorrect depending on the system and functional. D4 and D3(BJ) yield similar *D*_e_ MADs in the order of 0.96–5.82 kcal mol^−1^ and 0.80–5.83 kcal mol^−1^, respectively. For the same underlying functional, the two iterations of DFT-D yield comparable MADs where differences between D4 and D3(BJ) MADs do not exceed 0.02 Å or 1.16 kcal mol^−1^.

Dispersion-corrected global double hybrids give the best description of excimer binding with greatly improved *r*_e_ (ranging from 0.05 to 0.07 Å) and the smallest deviations in *D*_e_ despite their tendency to overbind the excimer systems: their MADs for *D*_e_ range from 0.80 kcal mol^−1^ [B2GP-PLYP-D3(BJ)] to 1.85 kcal mol^−1^ [B2PLYP-D4]. Overestimation of exciplex binding from dispersion-corrected double hybrids was also noted in the study by Krueger and Blanquart where B2PLYP-D3(BJ) significantly overbound the exciplexes analysed which the authors acknowledged was largely due to the inclusion of a dispersion correction.^102^ Their tendency to overestimate *D*_e_ suggests that excited-state parametrised dispersion corrections would be necessary for a reliable description by TD-DFT methods.

Dispersion-corrected RS-DHDFAs only slightly improve on dispersion-uncorrected results, with MADs for *D*_e_ that range from 4.14 [ωB2GP-PLYP-D3(BJ)/D4] to 5.83 kcal mol^−1^ [ωB2PLYP-D3(BJ)]. MADs in *r*_e_ are unchanged from dispersion-uncorrected RS-DHFAs (0.04–0.05 Å) remaining the best performing across all functionals dispersion-corrected or otherwise. As the first application of ground-state parametrised dispersion corrections to RS-DHDFAs for excited-state problems, these functionals display potential for TD-DFT dissociation energies that approach SCS-CC2/CBS(3,4) quality. However, their tendency to underestimate *D*_e_ reinforces the necessity of excited-state parametrised dispersion corrections for a reliable description by TD-DFT methods.

From the discussion of dispersion-uncorrected functionals, double hybrids and range-separated double hybrids performed well for the more complicated excited-state interactions comprising excimer binding. As with range-separated double hybrids, the ground-state parametrised dispersion corrections do not reliably account for excited-state dispersion interactions, but without accounting for this missing dispersion TD-DFT cannot provide reliable results for excited states. While with current dispersion corrections both types of DHDFAs seem to be adequate, it is safe to assume that the better description of CT with RS-DHDFAs means that they will prevail once paired with state-specific corrections.

Global hybrids and CAM-B3LYP show a significant error reduction upon dispersion correction yielding MADs for *D*_e_ in the order of 2.15–4.36 kcal mol^−1^, more comparable to the MADs of higher-rung dispersion-corrected functionals, alongside an improved but still overestimated *r*_e_ with MADs ranging from 0.07 to 0.12 Å. The dispersion corrections offer considerable improvement to excimer binding for these less sophisticated functionals which gave the largest MADs when dispersion-uncorrected. However, even with dispersion correction, global hybrids and CAM-B3LYP are still consistently outperformed by DHDFAs.

## 5 Summary and conclusion

The binding of four different aromatic excimer models was analysed by means of dissociation curves. To our knowledge, this is the first study to provide single-reference wave function curves at the complete basis set (CBS) limit for aromatic excimer systems. More specifically, linear SCS-CC2/CBS(3,4) was established as a reliable reference that allowed us to shed light onto various TD-DFT methods including a detailed analysis of excimers with double-hybrid density functionals and the first application of range-separated double hybrids to such systems. Our main goal was to address the impact of Fock exchange, range separation, the perturbative nonlocal correction used in double hybrids, and London dispersion corrections. We analysed two quantities that characterise the minima along the dissociation curves, namely dissociation energies *D*_e_ and inter-monomer equilibrium distances *r*_e_.

Overall, it turned out to be challenging to obtain a good description of both *D*_e_ and *r*_e_ with hybrid functionals, whereas double hybrids provided a more robust picture. We found no ideal admixture of Fock exchange in global hybrids as its increase led to smaller dissociation energies and larger equilibrium distances, with large errors across both quantities. Dispersion-uncorrected global and range-separated hybrids, as well as global and range-separated double hybrids, all gave curves with distinct minima, with the latter functional type giving the best curves, most likely due to a better description of charge-transfer and exciton coupling. That being said, all dispersion-uncorrected TD-DFT methods produced large errors that increased considerably with system size. For the smallest system, the benzene excimer, dispersion-uncorrected methods see the best results from range-separated double hybrids, approaching the accuracy of SCS-CC2/CBS(3,4) quality. However, for larger systems there was greater disparity between even the most accurate TD-DFT methods and the reference. *D*_e_ values were usually underestimated and *r*_e_ values overestimated, which points to missing dispersion interactions as the likely reason.

The application of ground-state parametrised dispersion corrections generally reduced the errors of dispersion-uncorrected functionals but did not reach chemical accuracy most likely due to not having been designed to describe excited states. We have noticed overstabilisation of some systems for some functionals, which occurred more often for the smaller systems. For some methods and systems, improvements of the minimum-energy regions were observed, but dissociation energies were still underestimated. To our knowledge, we were also the first to point out that most TD-DFT methods were unphysically repulsive in the mid range, something that could be reduced by applying dispersion corrections, which in turn indicated that dispersion was the most dominant contribution in that range.

Our study has shown that some of the latest and most modern TD-DFT methods, namely range-separated double hybrids, belong to the most robust and accurate when treating excited states, which parallels single-molecule studies.^52,53,62–64,66^ However, we have also shown that there is a need for the development of state-specific London dispersion corrections to achieve a reliable and robust TD-DFT description of excimers and related systems for all tested methods, including range-separated double hybrids.

## Conflicts of interest

There are no conflicts of interest to declare.

## Supplementary Material

RA-013-D3RA07381E-s001

RA-013-D3RA07381E-s002

## References

[cit1] Turro N. J. , Modern Molecular Photochemistry, University Science Books, Sausalito, 1991

[cit2] Birks J. B. (1975). Rep. Prog. Phys..

[cit3] Saigusa H., Lim E. C. (1996). Acc. Chem. Res..

[cit4] Förster T. (1969). Angew. Chem., Int. Ed..

[cit5] Huenerbein R., Grimme S. (2008). Chem. Phys..

[cit6] Heissenbüttel M. C., Marauhn P., Deilmann T., Krüger P., Rohlfing M. (2019). Phys. Rev. B.

[cit7] Shirai S., Iwata S., Tani T., Inagaki S. (2011). J. Phys. Chem. A.

[cit8] Ge Q., Head-Gordon M. (2018). J. Chem. Theory Comput..

[cit9] Ge Q., Mao Y., Head-Gordon M. (2018). J. Chem. Phys..

[cit10] Förster T., Kasper K. (1955). Z. Elektrochem..

[cit11] Dover C. B., Gallaher J. K., Frazer L., Tapping P. C., Petty A. J., Crossley M. J., Anthony J. E., Kee T. W., Schmidt T. W. (2018). Nat. Chem..

[cit12] Sung J., Nowak-Król A., Schlosser F., Fimmel B., Kim W., Kim D., Würthner F. (2016). J. Am. Chem. Soc..

[cit13] Ye T., Singh R., Butt H. J., Floudas G., Keivanidis P. E. (2013). ACS Appl. Mater. Interfaces.

[cit14] Thirion D., Romain M., Rault-Berthelot J., Poriel C. (2012). J. Mater. Chem..

[cit15] Vollbrecht J. (2018). New J. Chem..

[cit16] Thirupathi P., Park J.-Y., Neupane L. N., Kishore M. Y. L. N., Lee K.-H. (2015). ACS Appl. Mater. Interfaces.

[cit17] Focsaneanu K.-S., Scaiano J. C. (2005). Photochem. Photobiol. Sci..

[cit18] Østergaard M. E., Hrdlicka P. J. (2011). Chem. Soc. Rev..

[cit19] Karuppannan S., Chambron J.-C. (2011). Chem.–Asian J..

[cit20] Basting D. and Marowsky G., Excimer laser technology, Springer, 2005

[cit21] Ibele L. M., Sánchez-Murcia P. A., Mai S., Nogueira J. J., González L. (2020). J. Phys. Chem. Lett..

[cit22] Improta R. (2014). Chem.–Eur. J..

[cit23] Hohenberg P., Kohn W. (1964). Phys. Rev..

[cit24] Kohn W., Sham L. J. (1965). Phys. Rev..

[cit25] Goerigk L., Grimme S. (2011). Phys. Chem. Chem. Phys..

[cit26] Mardirossian N., Head-Gordon M. (2017). Mol. Phys..

[cit27] Goerigk L., Hansen A., Bauer C., Ehrlich S., Najibi A., Grimme S. (2017). Phys. Chem. Chem. Phys..

[cit28] Mehta N., Casanova-Páez M., Goerigk L. (2018). Phys. Chem. Chem. Phys..

[cit29] Najibi A., Goerigk L. (2018). J. Chem. Theory Comput..

[cit30] Santra G., Sylvetsky N., Martin J. M. L. (2019). J. Phys. Chem. A.

[cit31] Najibi A., Goerigk L. (2020). J. Comput. Chem..

[cit32] Kristyán S., Pulay P. (1994). Chem. Phys. Lett..

[cit33] Pérez-Jordá J., Becke A. D. (1995). Chem. Phys. Lett..

[cit34] Hobza P., Šponer J., Reschel T. (1995). J. Comput. Chem..

[cit35] Šponer J., Leszczynski J., Hobza P. (1996). J. Comput. Chem..

[cit36] Goerigk L. (2014). J. Chem. Theory Comput..

[cit37] Goerigk L. (2015). J. Phys. Chem. Lett..

[cit38] Mardirossian N., Head-Gordon M. (2016). J. Chem. Theory Comput..

[cit39] Vydrov O. A., Van Voorhis T. (2010). J. Chem. Phys..

[cit40] Grimme S., Ehrlich S., Goerigk L. (2011). J. Comput. Chem..

[cit41] Dobson J. F. (2014). Int. J. Quantum Chem..

[cit42] Grimme S., Hansen A., Brandenburg J. G., Bannwarth C. (2016). Chem. Rev..

[cit43] Goerigk L., Mehta N. (2019). Aust. J. Chem..

[cit44] Morgante P., Peverati R. (2020). Int. J. Quantum Chem..

[cit45] Martin J. M. L., Santra G. (2020). Isr. J. Chem..

[cit46] Grimme S. (2006). J. Chem. Phys..

[cit47] Goerigk L., Grimme S. (2014). Wiley Interdiscip. Rev.: Comput. Mol. Sci..

[cit48] Gross E. K. U., Kohn W. (1990). Adv. Quantum Chem..

[cit49] Casida M. E. , in Time-Dependent Density Functional Response Theory for Molecules, World Scientific Singapore, 1995, pp. 155–192

[cit50] Bauernschmitt R., Ahlrichs R. (1996). Chem. Phys. Lett..

[cit51] Schwabe T., Goerigk L. (2017). J. Chem. Theory Comput..

[cit52] Casanova-Páez M., Dardis M. B., Goerigk L. (2019). J. Chem. Theory Comput..

[cit53] Casanova-Páez M., Goerigk L. (2021). J. Chem. Theory Comput..

[cit54] Goerigk L., Grimme S. (2009). J. Phys. Chem. A.

[cit55] Goerigk L., Moellmann J., Grimme S. (2009). Phys. Chem. Chem. Phys..

[cit56] Jacquemin D., Wathelet V., Perpète E. A., Adamo C. (2009). J. Chem. Theory Comput..

[cit57] Goerigk L., Grimme S. (2010). J. Chem. Phys..

[cit58] Goerigk L., Grimme S. (2011). J. Chem. Theory Comput..

[cit59] Leang S. S., Zahariev F., Gordon M. S. (2012). J. Chem. Phys..

[cit60] Laurent A. D., Jacquemin D. (2013). Int. J. Quantum Chem..

[cit61] Brémond E., Savarese M., Adamo C., Jacquemin D. (2018). J. Chem. Theory Comput..

[cit62] Casanova-Páez M., Goerigk L. (2020). J. Chem. Phys..

[cit63] Casanova-Páez M., Goerigk L. (2021). J. Comput. Chem..

[cit64] Van Dijk J., Casanova-Páez M., Goerigk L. (2022). ACS Phys. Chem. Au.

[cit65] Perdew J. P., Schmidt K. (2001). AIP Conf. Proc..

[cit66] Goerigk L., Casanova-Paéz M. (2021). Aust. J. Chem..

[cit67] Tozer D. J., Amos R. D., Handy N. C., Roos B. O., Serrano-Andres L. (1999). Mol. Phys..

[cit68] Tozer D. J. (2003). J. Chem. Phys..

[cit69] Dreuw A., Weisman J. L., Head-Gordon M. (2003). J. Chem. Phys..

[cit70] Dreuw A., Head-Gordon M. (2004). J. Am. Chem. Soc..

[cit71] Maitra N. T. (2017). J. Phys.: Condens. Matter.

[cit72] Leininger T., Stoll H., Werner H.-J., Savin A. (1997). Chem. Phys. Lett..

[cit73] Iikura H., Tsuneda T., Yanai T., Hirao K. (2001). J. Chem. Phys..

[cit74] Yanai T., Tew D. P., Handy N. C. (2004). Chem. Phys. Lett..

[cit75] Baer R., Neuhauser D. (2005). Phys. Rev. Lett..

[cit76] Richard R. M., Herbert J. M. (2011). J. Chem. Theory Comput..

[cit77] Grimme S., Neese F. (2007). J. Chem. Phys..

[cit78] Head-Gordon M., Rico R. J., Oumi M., Lee T. J. (1994). Chem. Phys. Lett..

[cit79] Goerigk L. , Kruse H. and Grimme S., in Theoretical Electronic Circular Dichroism Spectroscopy of Large Organic and Supramolecular Systems, Wiley-Blackwell, 2012, ch. 22, pp. 643–673

[cit80] Hirata S., Head-Gordon M. (1999). Chem. Phys. Lett..

[cit81] Mester D., Kállay M. (2021). J. Chem. Theory Comput..

[cit82] Mester D., Kállay M. (2021). J. Chem. Theory Comput..

[cit83] Mester D., Kállay M. (2022). J. Chem. Theory Comput..

[cit84] Grimme S. (2006). J. Comput. Chem..

[cit85] Krueger R. A., Blanquart G. (2019). Phys. Chem. Chem. Phys..

[cit86] Velardez G. F., Lemke H. T., Breiby D. W., Nielsen M. M., Møller K. B., Henriksen N. E. (2008). J. Phys. Chem. A.

[cit87] Peverati R., Truhlar D. G. (2014). Philos. Trans. R. Soc., A.

[cit88] Casanova D. (2015). Int. J. Quantum Chem..

[cit89] Goerigk L., Kruse H., Grimme S. (2011). ChemPhysChem.

[cit90] Kołaski M., Arunkumar C. R., Kim K. S. (2013). J. Chem. Theory Comput..

[cit91] Capello M. C., Hernández F. J., Broquier M., Dedonder-Lardeux C., Jouvet C., Pino G. A. (2016). Phys. Chem. Chem. Phys..

[cit92] Dubinets N. O., Safonov A. A., Bagaturyants A. A. (2016). J. Phys. Chem. A.

[cit93] Hoche J., Schmitt H. C., Humeniuk A., Fischer I., Mitrić R., Röhr M. I. (2017). Phys. Chem. Chem. Phys..

[cit94] Safonov A. A., Bagaturyants A. A., Sazhnikov V. A. (2017). J. Mol. Model..

[cit95] Kokkin D., Ivanov M., Loman J., Cai J.-Z., Uhler B., Reilly N., Rathore R., Reid S. A. (2018). J. Chem. Phys..

[cit96] Grimme S., Antony J., Ehrlich S., Krieg H. (2010). J. Chem. Phys..

[cit97] Becke A. D., Johnson E. R. (2005). J. Chem. Phys..

[cit98] Johnson E. R., Becke A. D. (2005). J. Chem. Phys..

[cit99] Johnson E. R., Becke A. D. (2006). J. Chem. Phys..

[cit100] Elfers N., Lyskov I., Spiegel J. D., Marian C. M. (2016). J. Phys. Chem. C.

[cit101] Anger I., Rykova E., Bagaturyants A. (2017). ChemistrySelect.

[cit102] Krueger R. A., Blanquart G. (2019). J. Phys. Chem. A.

[cit103] Ye C., Gray V., Kushwaha K., Kumar Singh S., Erhart P., Börjesson K. (2020). Phys. Chem. Chem. Phys..

[cit104] Gao Y., Liu H., Zhang S., Gu Q., Shen Y., Ge Y., Yang B. (2018). Phys. Chem. Chem. Phys..

[cit105] do Casal M. T., Cardozo T. M. (2020). Theor. Chem. Acc..

[cit106] Diaz-Andres A., Casanova D. (2021). J. Phys. Chem. Lett..

[cit107] Ikabata Y., Nakai H. (2012). J. Chem. Phys..

[cit108] Sato T., Nakai H. (2009). J. Chem. Phys..

[cit109] Sato T., Nakai H. (2010). J. Chem. Phys..

[cit110] Řezáč J., Riley K. E., Hobza P. (2011). J. Chem. Theory Comput..

[cit111] Briggs E. A., Besley N. A. (2014). Phys. Chem. Chem. Phys..

[cit112] Becke A. D., Johnson E. R. (2007). J. Chem. Phys..

[cit113] Feng X., Otero-de-la Roza A., Johnson E. R. (2018). Can. J. Chem..

[cit114] Barone V., Biczysko M., Pavone M. (2008). Chem. Phys..

[cit115] Fabrizio A., Corminboeuf C. (2018). J. Phys. Chem. Lett..

[cit116] Amicangelo J. C. (2005). J. Phys. Chem. A.

[cit117] Shi B., Nachtigallová D., Aquino A. J., Machado F. B., Lischka H. (2019). J. Phys. Chem. Lett..

[cit118] Shirai S., Kurashige Y., Yanai T. (2016). J. Chem. Theory Comput..

[cit119] Reiter S., Roos M. K., Vivie-Riedle R. (2019). ChemPhotoChem.

[cit120] Rocha-Rinza T., Christiansen O. (2009). Chem. Phys. Lett..

[cit121] Rocha-Rinza T., De Vico L., Veryazov V., Roos B. O. (2006). Chem. Phys. Lett..

[cit122] Caldeweyher E., Bannwarth C., Grimme S. (2017). J. Chem. Phys..

[cit123] Caldeweyher E., Ehlert S., Hansen A., Neugebauer H., Spicher S., Bannwarth C., Grimme S. (2019). J. Chem. Phys..

[cit124] Furche F., Ahlrichs R., Hättig C., Klopper W., Sierka M., Weigend F. (2014). Wiley Interdiscip. Rev.: Comput. Mol. Sci..

[cit125] Häser M., Ahlrichs R. (1989). J. Comput. Chem..

[cit126] Hättig C., Weigend F. (2000). J. Chem. Phys..

[cit127] Grimme S. (2003). J. Chem. Phys..

[cit128] Grimme S., Goerigk L., Fink R. F. (2012). Wiley Interdiscip. Rev.: Comput. Mol. Sci..

[cit129] Hellweg A., Grün S. A., Hättig C. (2008). Phys. Chem. Chem. Phys..

[cit130] Weigend F., Ahlrichs R. (2005). Phys. Chem. Chem. Phys..

[cit131] Christiansen O., Koch H., Jørgensen P. (1995). Chem. Phys. Lett..

[cit132] Casanova D., Rhee Y. M., Head-Gordon M. (2008). J. Chem. Phys..

[cit133] Kim D. (2014). Bull. Korean Chem. Soc..

[cit134] Vahtras O., Almlöf J., Feyereisen M. (1993). Chem. Phys. Lett..

[cit135] Weigend F., Köhn A., Hättig C. (2002). J. Chem. Phys..

[cit136] Christiansen O., Koch H., Jørgensen P. (1996). J. Chem. Phys..

[cit137] Aidas K., Angeli C., Bak K. L., Bakken V., Bast R., Boman L., Christiansen O., Cimiraglia R., Coriani S., Dahle P., Dalskov E. K., Ekström U., Enevoldsen T., Eriksen J. J., Ettenhuber P., Fernández B., Ferrighi L., Fliegl H., Frediani L., Hald K., Halkier A., Hättig C., Heiberg H., Helgaker T., Hennum A. C., Hettema H., Hjertenæs E., Høst S., Høyvik I. M., Iozzi M. F., Jansík B., Jensen H. J. A., Jonsson D., Jørgensen P., Kauczor J., Kirpekar S., Kjærgaard T., Klopper W., Knecht S., Kobayashi R., Koch H., Kongsted J., Krapp A., Kristensen K., Ligabue A., Lutnæs O. B., Melo J. I., Mikkelsen K. V., Myhre R. H., Neiss C., Nielsen C. B., Norman P., Olsen J., Olsen J. M. H., Osted A., Packer M. J., Pawlowski F., Pedersen T. B., Provasi P. F., Reine S., Rinkevicius Z., Ruden T. A., Ruud K., Rybkin V. V., Sałek P., Samson C. C., de Merás A. S., Saue T., Sauer S. P., Schimmelpfennig B., Sneskov K., Steindal A. H., Sylvester-Hvid K. O., Taylor P. R., Teale A. M., Tellgren E. I., Tew D. P., Thorvaldsen A. J., Thøgersen L., Vahtras O., Watson M. A., Wilson D. J., Ziolkowski M., Ågren H. (2014). Wiley Interdiscip. Rev.: Comput. Mol. Sci..

[cit138] Perdew J. P., Burke K., Ernzerhof M. (1996). Phys. Rev. Lett..

[cit139] Becke A. D. (1988). Phys. Rev. A.

[cit140] Lee C., Yang W., Parr R. G. (1988). Phys. Rev. B: Condens. Matter Mater. Phys..

[cit141] Miehlich B., Savin A., Stoll H., Preuss H. (1989). Chem. Phys. Lett..

[cit142] Becke A. D. (1993). J. Chem. Phys..

[cit143] Stephens P. J., Devlin F. J., Chabalowski C. F., Frisch M. J. (1994). J. Phys. Chem..

[cit144] Becke A. D. (1993). J. Chem. Phys..

[cit145] Chai J.-D., Head-Gordon M. (2008). J. Chem. Phys..

[cit146] Karton A., Tarnopolsky A., Lamére J.-F., Schatz G. C., Martin J. M. L. (2008). J. Phys. Chem. A.

[cit147] Neese F. (2012). Wiley Interdiscip. Rev.: Comput. Mol. Sci..

[cit148] Neese F. (2018). Wiley Interdiscip. Rev.: Comput. Mol. Sci..

[cit149] Grimme S. , DFT-D3 V3.1, University of Bonn, 2015

[cit150] Caldeweyher E. , Ehlert S. and Grimme S., DFT-D4 Version 2.0, Mulliken Center for Theoretical Chemistry, University of Bonn, Germany, 2019

[cit151] Goerigk L., Grimme S. (2011). J. Chem. Theory Comput..

[cit152] Najibi A., Casanova-Páez M., Goerigk L. (2021). J. Phys. Chem. A.

[cit153] Santra G., Martin J. M. (2021). J. Chem. Theory Comput..

[cit154] Bartlett R. J., Musiał M. (2007). Rev. Mod. Phys..

[cit155] Izsák R. (2020). Wiley Interdiscip. Rev.: Comput. Mol. Sci..

[cit156] Raghavachari K., Trucks G. W., Pople J. A., Head-Gordon M. (1989). Chem. Phys. Lett..

[cit157] Karton A. (2016). Wiley Interdiscip. Rev.: Comput. Mol. Sci..

[cit158] Jurečka P., Šponer J., Černý J., Hobza P. (2006). Phys. Chem. Chem. Phys..

[cit159] Řezáč J., Hobza P. (2013). J. Chem. Theory Comput..

[cit160] Mehta N., Fellowes T., White J. M., Goerigk L. (2021). J. Chem. Theory Comput..

[cit161] Koch H., Christiansen O., Jørgensen P., Sanchez De Merás A. M., Helgaker T. (1997). J. Chem. Phys..

[cit162] Sauer S. P., Schreiber M., Silva-Junior M. R., Thiel W. (2009). J. Chem. Theory Comput..

[cit163] Loos P. F., Scemama A., Blondel A., Garniron Y., Caffarel M., Jacquemin D. (2018). J. Chem. Theory Comput..

[cit164] Loos P.-F., Scemama A., Boggio-Pasqua M., Jacquemin D. (2020). J. Chem. Theory Comput..

[cit165] Véril M., Scemama A., Caffarel M., Lipparini F., Boggio-Pasqua M., Jacquemin D., Loos P. F. (2021). Wiley Interdiscip. Rev.: Comput. Mol. Sci..

[cit166] Riplinger C., Sandhoefer B., Hansen A., Neese F. (2013). J. Chem. Phys..

[cit167] Riplinger C., Pinski P., Becker U., Valeev E. F., Neese F. (2016). J. Chem. Phys..

[cit168] Jensen F. , Introduction to Computational Chemistry, John Wiley & Sons, Chichester, 3rd edn, 2017

[cit169] Krueger R. A., Blanquart G. (2019). Int. J. Quantum Chem..

[cit170] Galano A., Alvarez-Idaboy J. R. (2006). J. Comput. Chem..

[cit171] Jensen F. (2010). J. Chem. Theory Comput..

[cit172] Kruse H., Grimme S. (2012). J. Chem. Phys..

[cit173] Otero-De-La-Roza A., DiLabio G. A. (2017). J. Chem. Theory Comput..

[cit174] Loos P. F., Pradines B., Scemama A., Giner E., Toulouse J. (2020). J. Chem. Theory Comput..

[cit175] Boys S. F., Bernardi F. (1970). Mol. Phys..

[cit176] Giner E., Scemama A., Toulouse J., Loos P.-F. (2019). J. Chem. Phys..

[cit177] Goerigk L., Grimme S. (2010). J. Chem. Theory Comput..

[cit178] Tajti A., Szalay P. G. (2019). J. Chem. Theory Comput..

[cit179] Winter N. O. C., Graf N. K., Leutwyler S., Hättig C. (2013). Phys. Chem. Chem. Phys..

[cit180] Schon C., Roth W., Fischer I., Pfister J., Kaiser C., Fink R. F., Engels B. (2010). Phys. Chem. Chem. Phys..

[cit181] Pfister J., Schon C., Roth W., Kaiser C., Lambert C., Gruss K., Braunschweig H., Fischer I., Fink R. F., Engels B. (2011). J. Phys. Chem. A.

[cit182] Rappoport D., Furche F. (2010). J. Chem. Phys..

[cit183] Dunning T. H. (1989). J. Chem. Phys..

[cit184] Kendall R. A., Dunning T. H., Harrison R. J. (1992). J. Chem. Phys..

[cit185] Papajak E., Zheng J., Xu X., Leverentz H. R., Truhlar D. G. (2011). J. Chem. Theory Comput..

[cit186] Karton A., Martin J. M. L. (2006). Theor. Chem. Acc..

[cit187] Neese F., Valeev E. F. (2011). J. Chem. Theory Comput..

[cit188] Halkier A., Helgaker T., Jørgensen P., Klopper W., Koch H., Olsen J., Wilson A. K. (1998). Chem. Phys. Lett..

[cit189] Kim D. (2017). Bull. Korean Chem. Soc..

[cit190] Sudhindra B. S., Chandra A. K. (1977). Curr. Sci..

[cit191] Stone A. , The Theory of Intermolecular Forces, Oxford University Press, 2nd edn, 2013

[cit192] Grimme S. (2004). J. Comput. Chem..

[cit193] Ruscic B. (2014). Int. J. Quantum Chem..

[cit194] Goerigk L., Grimme S. (2008). ChemPhysChem.

[cit195] Grimme S., Parac M. (2003). ChemPhysChem.

[cit196] Lin Y.-S., Li G.-D., Mao S.-P., Chai J.-D. (2013). J. Chem. Theory Comput..

[cit197] Loos P. F., Lipparini F., Boggio-Pasqua M., Scemama A., Jacquemin D. (2020). J. Chem. Theory Comput..

[cit198] Chai J.-D., Head-Gordon M. (2008). Phys. Chem. Chem. Phys..

[cit199] Mardirossian N., Head-Gordon M. (2014). Phys. Chem. Chem. Phys..

[cit200] Najibi A., Goerigk L. (2018). J. Chem. Theory Comput..

[cit201] Platt J. R. (1949). J. Chem. Phys..

[cit202] Parac M., Grimme S. (2003). Chem. Phys..

[cit203] Lopata K., Reslan R., Kowalska M., Neuhauser D., Govind N., Kowalski K. (2011). J. Chem. Theory Comput..

