# Supplementary material for: Noncovalently bound excited-state dimers: a perspective on current time-dependent density functional theory approaches applied to aromatic excimer models
Source: RSC Adv. 2023 Dec 11;13(51):35964–84. doi: 10.1039/d3ra07381e (PMC10712016; doi:10.1039/d3ra07381e)
Supplement: RA-013-D3RA07381E-s001 [file RA-013-D3RA07381E-s001.pdf]

Electronic Supplementary Material (ESI) for RSC Advances.
This journal is © The Royal Society of Chemistry 2023

Supporting Information: Noncovalently bound excited-state dimers: a perspective on current time-dependent Density Functional Theory approaches applied to aromatic excimer models

Amy C. Hancock and Lars Goerigk*

School of Chemistry, The University of Melbourne, Victoria 3010, Australia; Ph: +61-(0)3-83446784

E-mail: lars.goerigk@unimelb.edu.au

Contents

SI.1 Explicit definition of coordinates defining inter-monomer distance **S-3**

SI.2 Additional Data for Establishing Suitable Wavefunction Reference Method **S-3**
SI.2.1 Numerical Data for Wavefunction Methods S-3
SI.2.2 SCS-CC2 Dissociation Curves Comparing Basis Sets and Extrapolation to Complete Basis Set Limit S-4

SI.3 The ωB97X Problem **S-6**

SI.4 Additional Data For Benchmarking TD-DFT methods **S-7**
SI.4.1 Fock Exchange Study S-7
SI.4.1.1 Tables of Minima and Signed Percentage Errors S-7
SI.4.1.2 Dissociation Curves S-8
SI.4.2 DFT-D Corrected TD-DFT S-10
SI.4.2.1 Dissociation Curves S-10
SI.4.2.2 Unphysical Positive Regions in Mid-range of Dissociation Curves S-12
SI.4.3 Numerical Data For Binding Minima and Associated Percentage errors S-15
SI.4.4 Mean Absolute Deviations S-17

References **S-18**

SI.1 Explicit definition of coordinates defining inter-monomer distance

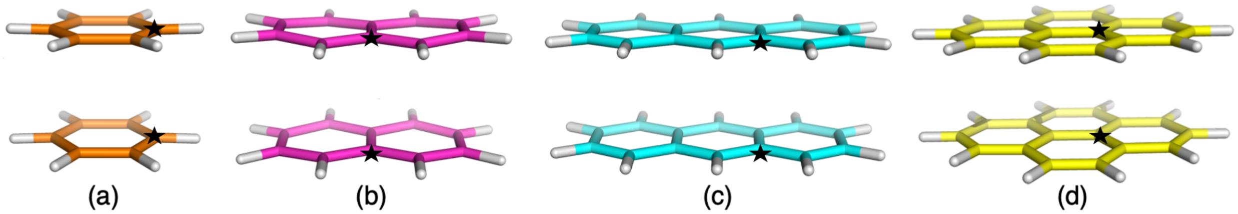

Figure S1: Diagrams of excimer model structures illustrating the atom coordinates used to define the inter-monomer distance for dissociation energy curves. Z-matrix templates used for defining the inter-monomer distance from these coordinates is given in the other supplementary files. Refer to README file for usage.

SI.2 Additional Data for Establishing Suitable Wavefunction Reference Method

SI.2.1 Numerical Data for Wavefunction Methods

Table S1: Explicit minima of SCS-CC2 and CC2 interaction energy curves of the lowest singlet excited state of the stacked benzene dimer for the basis set study. The distance of the minimum is denoted r_e (Å) and the associated interaction energy at that position ΔE (kcal/mol).

	SCS-CC2		CC2	
Basis set	r_e	ΔE	r_e	ΔE
def2-SVP	2.99	-12.60	2.90	-18.22
def2-TZVP	2.99	-13.18	2.90	-20.19
def2-QZVP	2.99	-13.29	2.90	-20.64
cc-pVTZ	3.00	-12.99	2.90	-19.95
def2-TZVPD	3.00	-15.61	2.90	-22.75
aug-cc-pVTZ	3.00	-15.03	2.90	-22.37

Table S2: CCSDR(3) and SCS-CC2 interaction energies (kcal/mol) of the lowest singlet excited state of the stacked benzene dimer relative to the excited state asymptote (value at 16 Å) for Karlsruhe basis sets and CBS limit extrapolations.

	CCSDR(3)			SCS-CC2	
Distance (Å)	def2-SVP	def2-TZVP	CBS(2,3)	CBS(2,3)	CBS(3,4)
2.90	-10.72	-11.31	-13.06	-14.56	-13.40
2.95	-11.19	-11.76	-13.41	-14.76	-13.63
3.00	-11.39	-11.96	-13.52	-14.73	-13.62
3.05	-11.36	-11.96	-13.42	-14.52	-13.42

SI.2.2 SCS-CC2 Dissociation Curves Comparing Basis Sets and Extrapolation to Complete Basis Set Limit

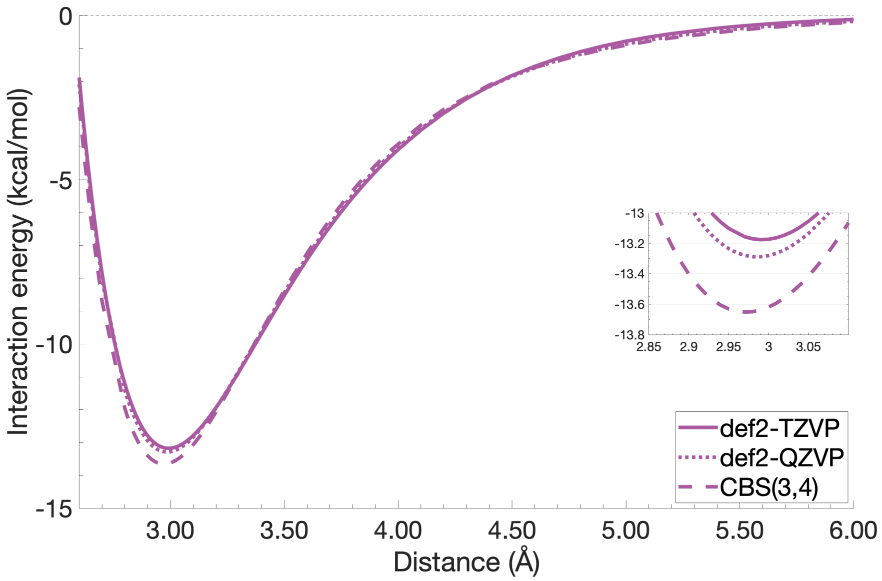

Figure S2: Dissociation curves for the lowest-lying excited state of the benzene excimer with SCS-CC2 across two successive basis sets in the Karlsruhe family (def2-nZVP) and their complete basis set extrapolated result, CBS(3,4). A magnified snapshot of the minima is shown to the right.

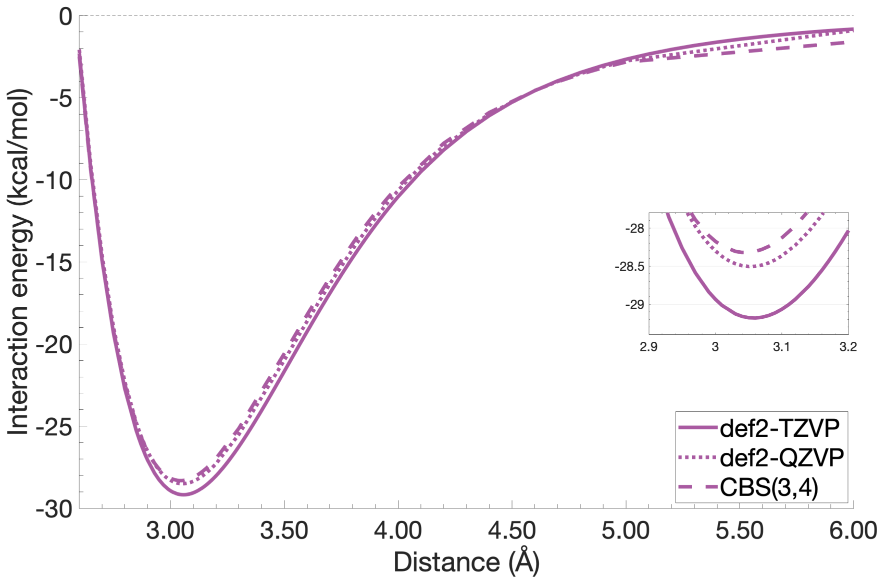

Figure S3: Dissociation curves for the lowest-lying excited state of the naphthalene excimer with spin-component scaled CC2 (SCS-CC2) across two successive basis sets in the Karlsruhe family (def2-nZVP) and their complete basis set extrapolated result, CBS(3,4). A magnified snapshot of the minima is shown to the right.

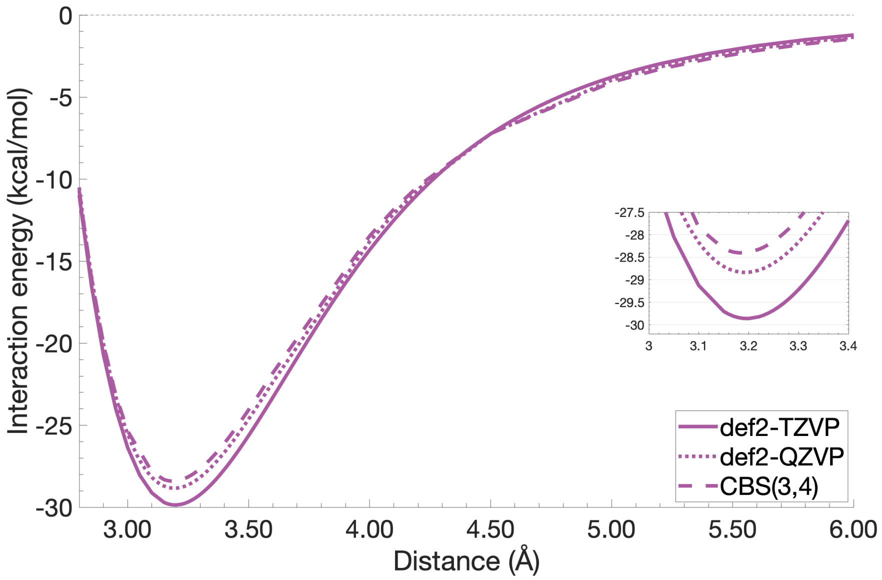

Figure S4: Dissociation curves for the lowest-lying excited state of the anthracene excimer with spin-component scaled CC2 (SCS-CC2) across two successive basis sets in the Karlsruhe family (def2-nZVP) and their complete basis set extrapolated result, CBS(3,4). A magnified snapshot of the minima is shown to the right.

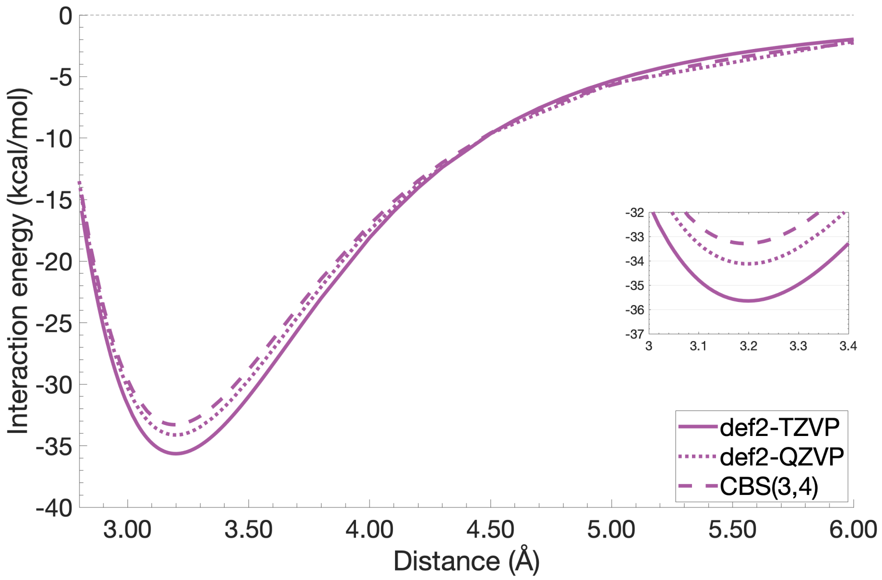

Figure S5: Dissociation curves for the lowest-lying excited state of the pyrene excimer with spin-component scaled CC2 (SCS-CC2) across two successive basis sets in the Karlsruhe family (def2-nZVP) and their complete basis set extrapolated result, CBS(3,4). A magnified snapshot of the minima is shown to the right.

SI.3 The ωB97X Problem

There were some unanticipated technical problems for ωB97X resulting in wobbly interaction energy curves as shown in the figures below.

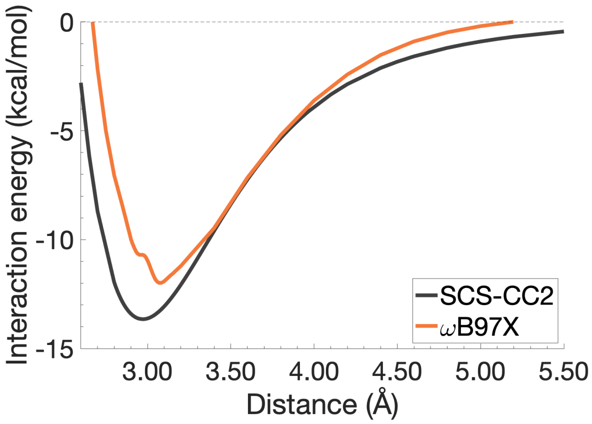

(a) Benzene excimer

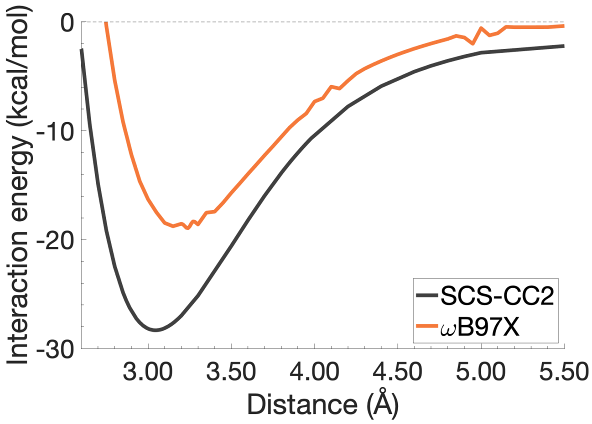

(b) Naphthalene excimer

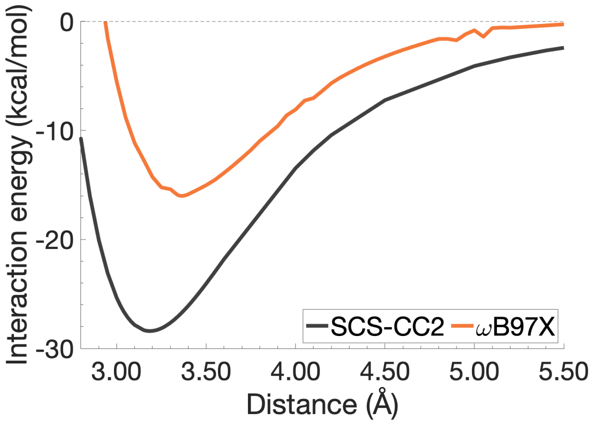

(c) Anthracene excimer

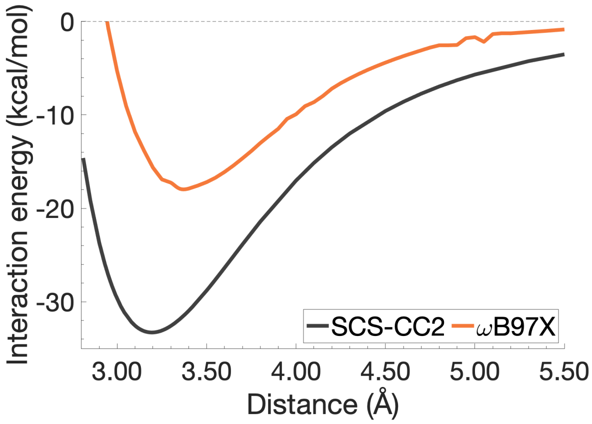

(d) Pyrene excimer

Figure S6: Dissociation energy curves for ωB97X and SCS-CC2/CBS(3,4) for each excimer. Due to the difficulty of identifying the actual minima for ωB97X we defined each D_e from the mathematical minimum of each curve.

SI.4 Additional Data For Benchmarking TD-DFT methods

SI.4.1 Fock Exchange Study

SI.4.1.1 Tables of Minima and Signed Percentage Errors

Table S3: Dissociation energies (D_e; kcal/mol), equilibrium inter-monomer distances (r_e; Å) and signed percentage errors relative to the SCS-CC2/CBS(3,4) reference for TD-BLYP and global hybrid variants with varied amounts of Fock exchange.

Excimer	Benzene	Naphthalene	Anthracene	Pyrene	Benzene	Naphthalene	Anthracene	Pyrene
Fock exchange (%)	D_e (kcal/mol)				r_e (Å)			
0^a	10.56	0.73	-	-	3.23	3.57	-	-
20	10.68	14.69	9.19	10.60	3.15	3.37	3.61	3.67
37.5	8.72	12.61	7.41	9.08	3.14	3.32	3.54	3.59
50	8.10	11.79	6.88	8.22	3.13	3.29	3.51	3.54
75	7.57	10.60	6.40	7.14	3.12	3.26	3.47	3.51
Fock exchange (%)	Error in D_e (%)				Error in r_e (%)			
0^a	-22.7	-97.4	-	-	8.8	17.4	-	-
20	-21.7	-48.1	-67.7	-68.2	6.1	10.9	13.5	15.0
37.5	-36.1	-55.5	-73.9	-72.7	5.7	9.2	11.3	12.5
50	-40.7	-58.4	-75.8	-75.3	5.4	8.2	10.4	11.0
75	-44.6	-62.6	-77.5	-78.6	5.1	7.2	9.1	10.0

^{a}BLYP does not predict minima for the anthracene and pyrene excimer states. See Fig. S7.

Table S4: Dissociation energies (D_e), equilibrium inter-monomer distances (r_e) and signed percentage errors relative to the SCS-CC2/CBS(3,4) reference for TD-PBE and global hybrid variants with varied amounts of Fock exchange.

Excimer	Benzene	Naphthalene	Anthracene	Pyrene	Benzene	Naphthalene	Anthracene	Pyrene
Fock exchange (%)	D_e (kcal/mol)				r_e (Å)			
0	15.38	5.42	3.23	1.76	3.10	3.36	3.60	3.71
20	14.65	18.99	13.24	14.44	3.05	3.25	3.47	3.52
37.5	12.62	17.00	11.31	13.09	3.03	3.20	3.40	3.44
50	11.50	15.69	10.10	11.53	3.02	3.17	3.38	3.41
75	9.92	13.27	7.98	8.69	3.00	3.15	3.35	3.38
Fock exchange (%)	Error in D_e (%)				Error in r_e (%)			
0	12.6	-80.9	-88.6	-94.7	4.4	10.5	13.2	16.3
20	7.3	-33.0	-53.4	-56.6	2.7	6.9	9.1	10.3
37.5	-7.6	-40.0	-60.2	-60.7	2.0	5.3	6.9	7.8
50	-15.7	-44.6	-64.4	-65.4	1.7	4.3	6.3	6.9
75	-27.3	-53.2	-71.9	-73.9	1.0	3.6	5.3	6.0

SI.4.1.2 Dissociation Curves

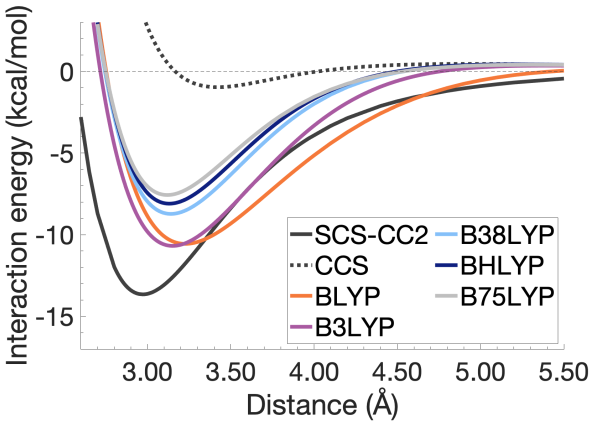

(a) Benzene excimer

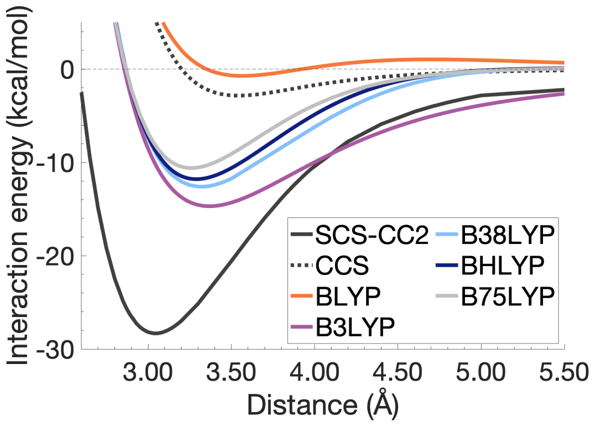

(b) Naphthalene excimer

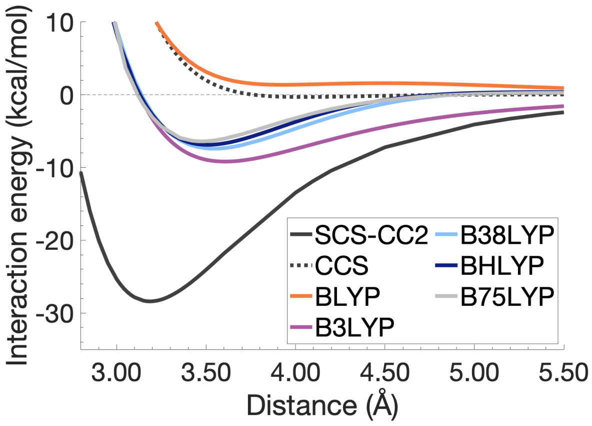

(c) Anthracene excimer

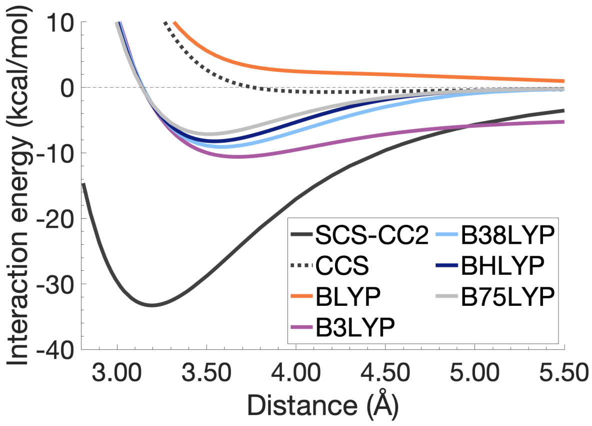

(d) Pyrene excimer

Figure S7: Dissociation energy curves for TD-BLYP and global hybrid variants with varied amounts of Fock exchange. SCS-CC2/CBS(3,4) and CCS/def2-TZVP curves are also shown for reference.

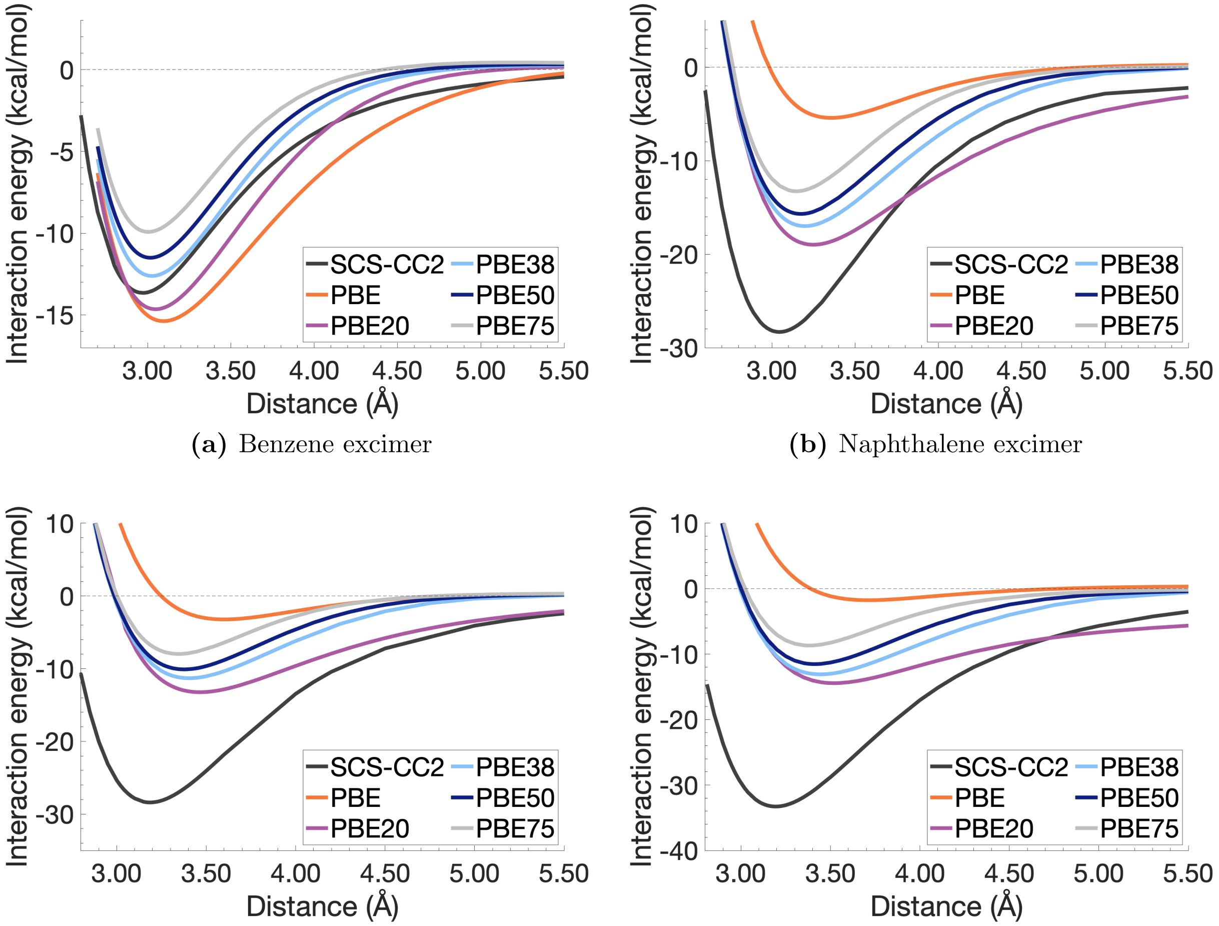

(a) Benzene excimer **(b)** Naphthalene excimer

(c) Anthracene excimer **(d)** Pyrene excimer

Figure S8: Dissociation energy curves for TD-PBE and global hybrid variants with varied amounts of Fock exchange. SCS-CC2/CBS(3,4) curves are also shown for reference.

SI.4.2 DFT-D Corrected TD-DFT

SI.4.2.1 Dissociation Curves

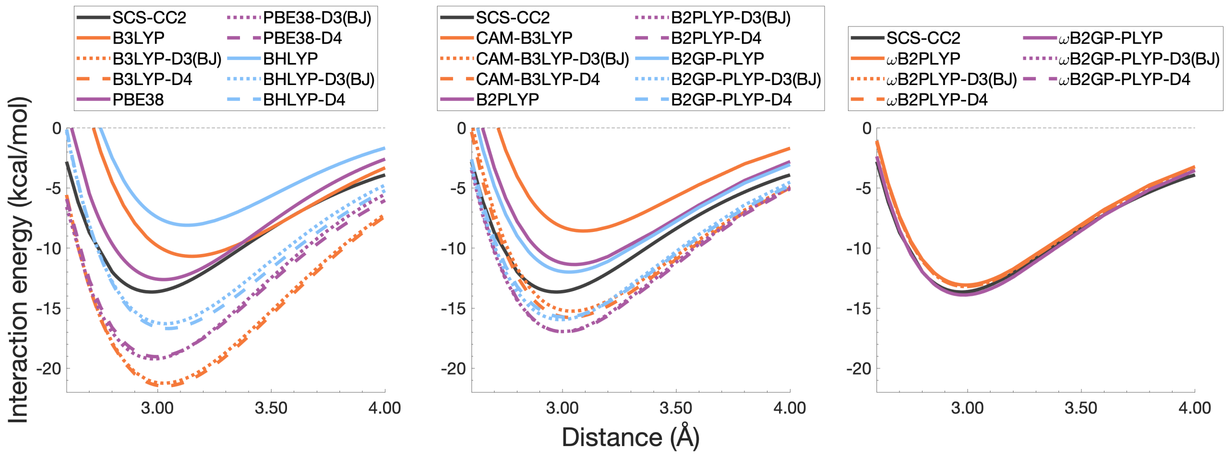

Figure S9: Dissociation energy curves for the benzene excimer comparing density functional approximations, with and without DFT-D type dispersion corrections, to the SCS-CC2/CBS(3,4) reference. All TD-DFT results are based on the def2-TZVP basis set.

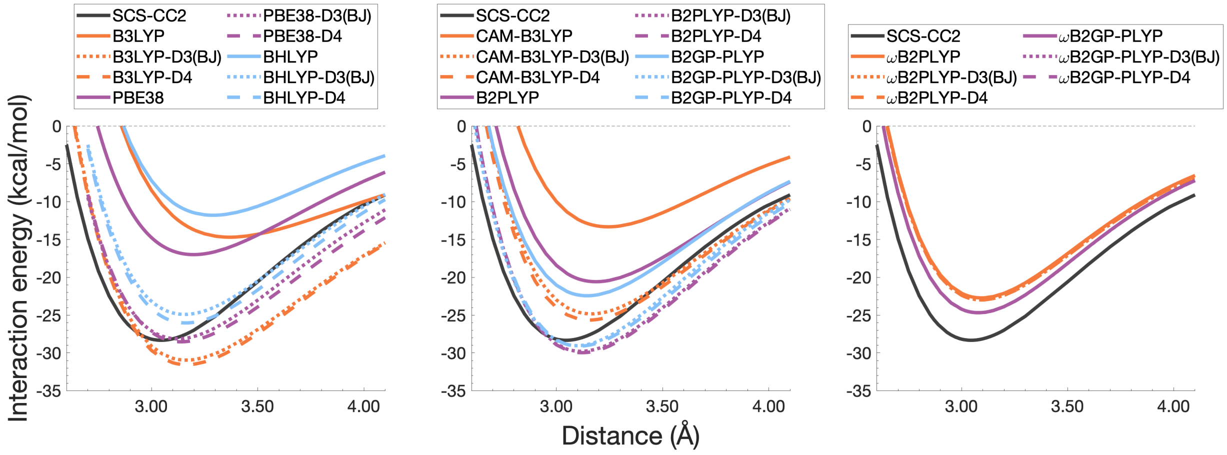

Figure S10: Dissociation energy curves for the naphthalene excimer comparing density functional approximations, with and without DFT-D type dispersion corrections, to the SCS-CC2/CBS(3,4) reference. All TD-DFT results are based on the def2-inter-monomerTZVP basis set.

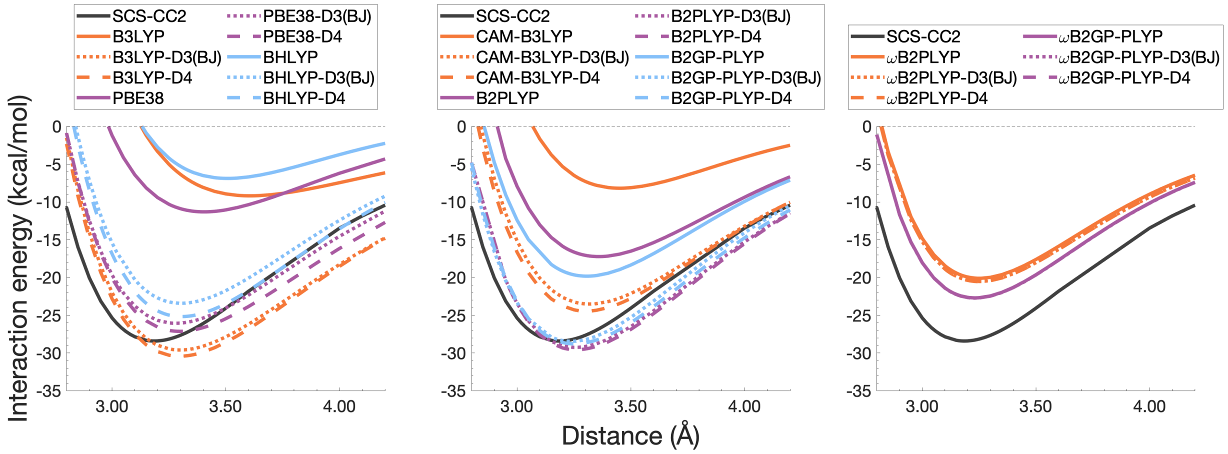

Figure S11: Dissociation energy curves for the anthracene excimer comparing density functional approximations, with and without DFT-D type dispersion corrections, to the SCS-CC2/CBS(3,4) reference.

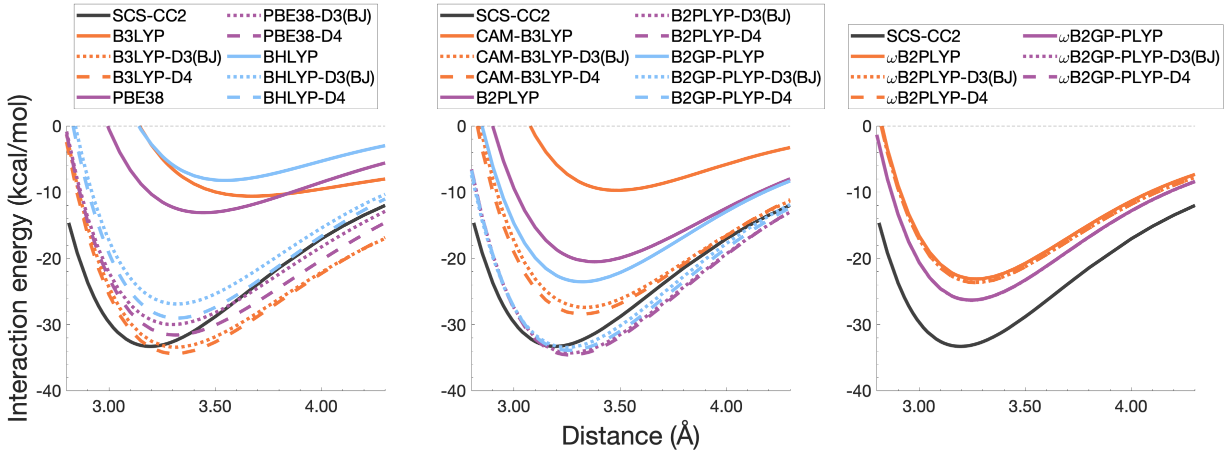

Figure S12: Dissociation energy curves for the pyrene excimer comparing density functional approximations, with and without DFT-D type dispersion corrections, to the SCS-CC2/CBS(3,4) reference.

SI.4.2.2 Unphysical Positive Regions in Mid-range of Dissociation Curves

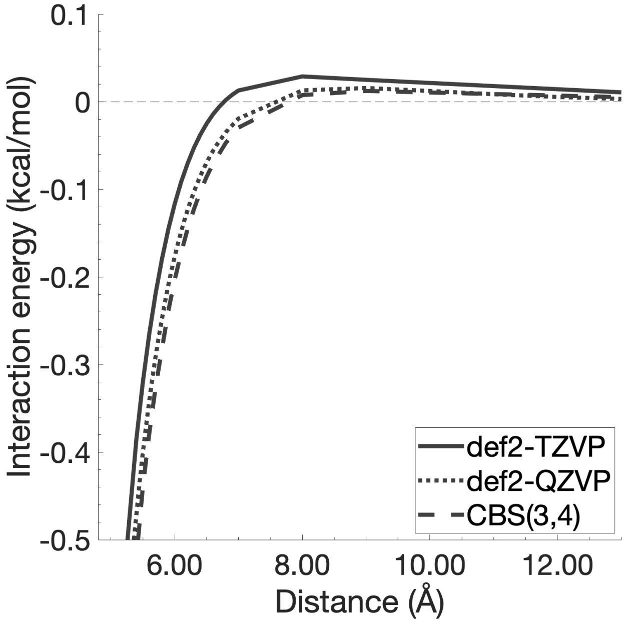

Figure S13: Mid-range region of SCS-CC2 dissociation curves for the benzene excimer with two truncated basis sets and their CBS(3,4) extrapolation.

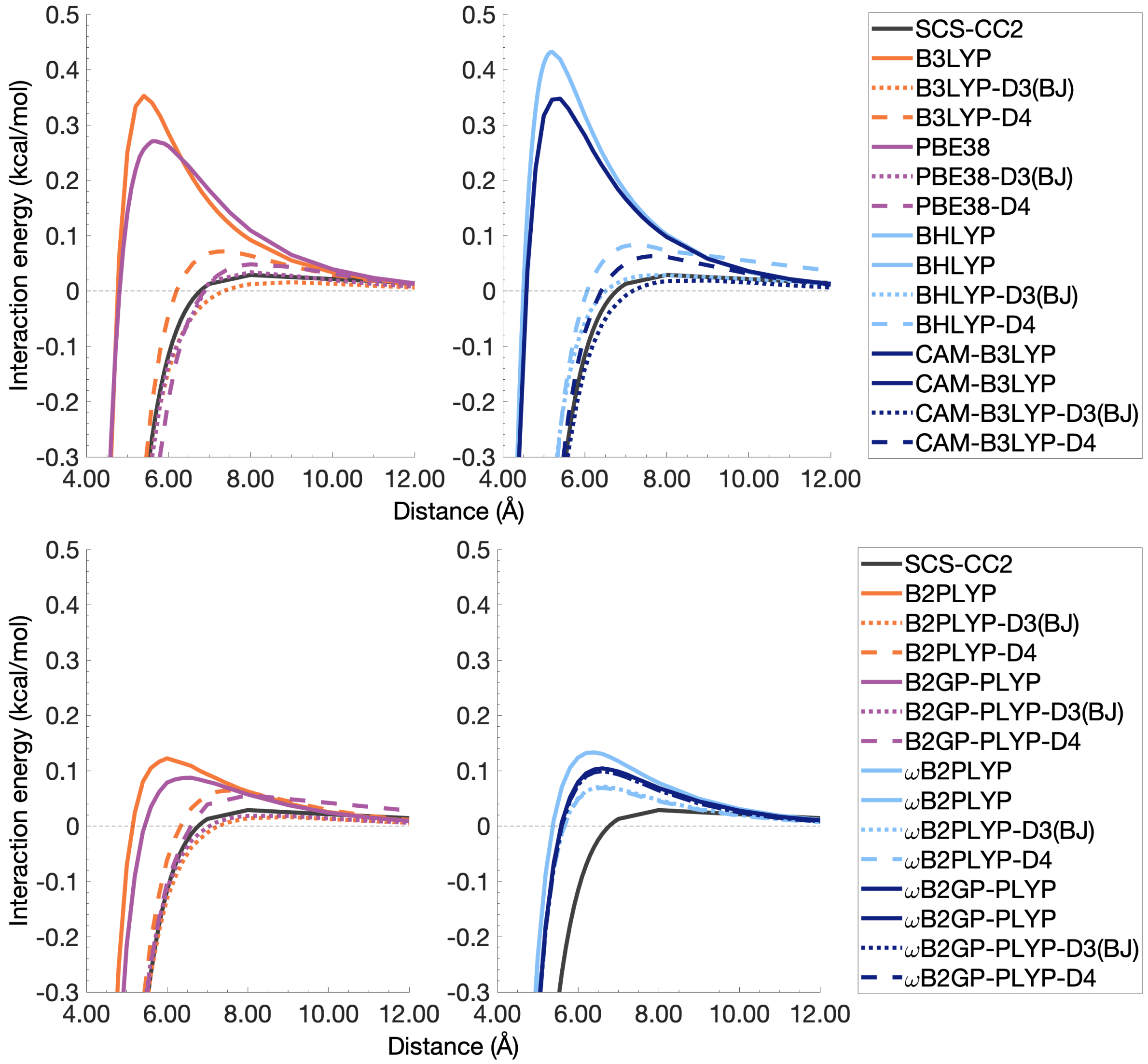

Figure S14: Mid-range region of the dissociation energy curves for the benzene excimer comparing density functional approximations, with and without DFT-D type dispersion corrections, to the SCS-CC2/CBS(3,4) reference.

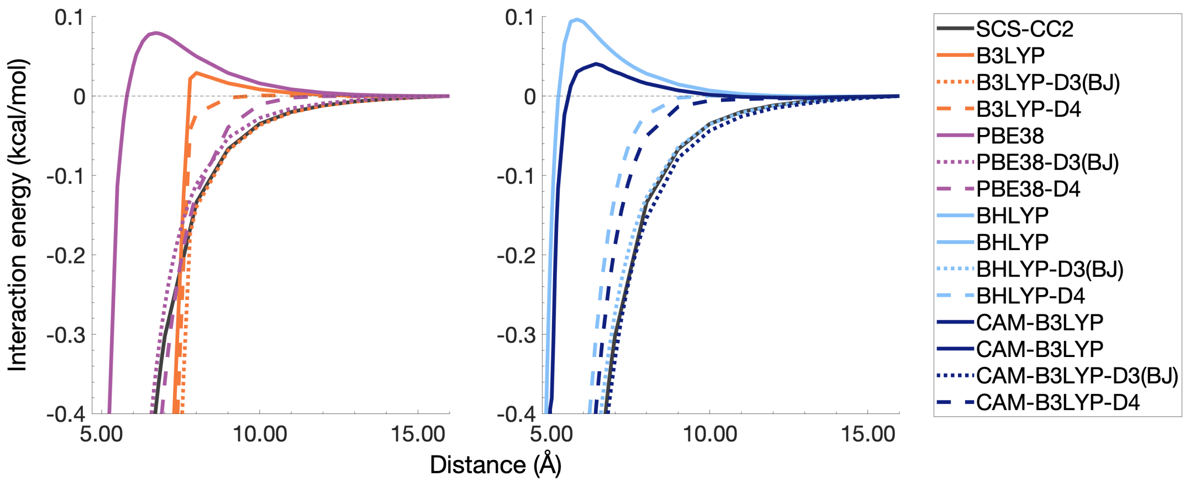

Figure S15: Mid-range region of the dissociation energy curves for the naphthalene excimer comparing density functional approximations, with and without DFT-D type dispersion corrections, to the SCS-CC2/def2-TZVP reference. All TD-DFT results are based on the def2-TZVP basis set. As distances beyond the binding region were not the focus of the paper, some curves may show strange shapes in the mid-range due to the small number of data points however they still showcase the identified positive region.

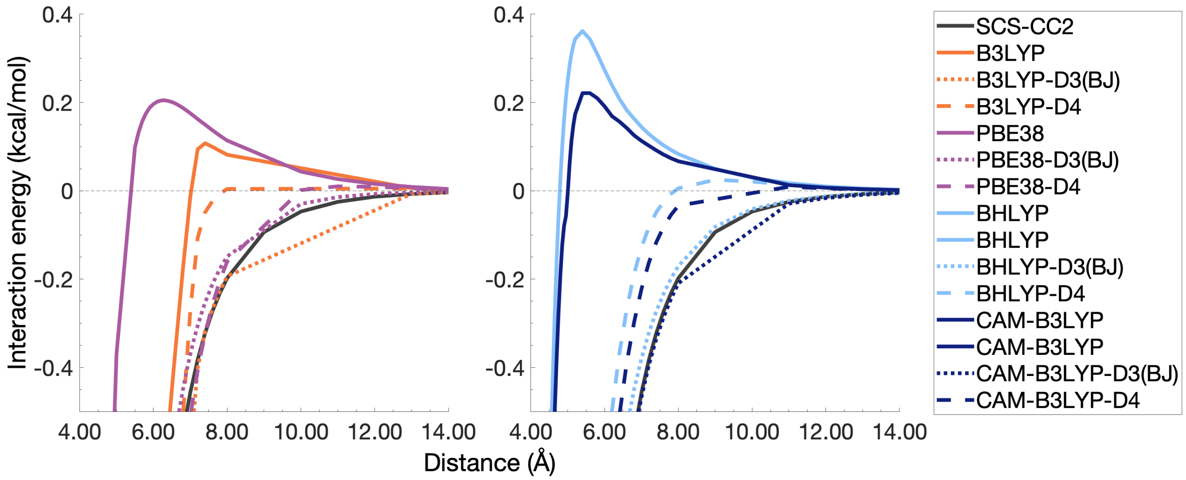

Figure S16: Mid-range region of the dissociation energy curves for the anthracene excimer comparing density functional approximations, with and without DFT-D type dispersion corrections, to the SCS-CC2/def2-TZVP reference. All TD-DFT results are based on the def2-TZVP basis set. As distances beyond the binding region were not the focus of the paper, some curves may show strange shapes in the mid-range due to the small number of data points however they still showcase the identified positive region.

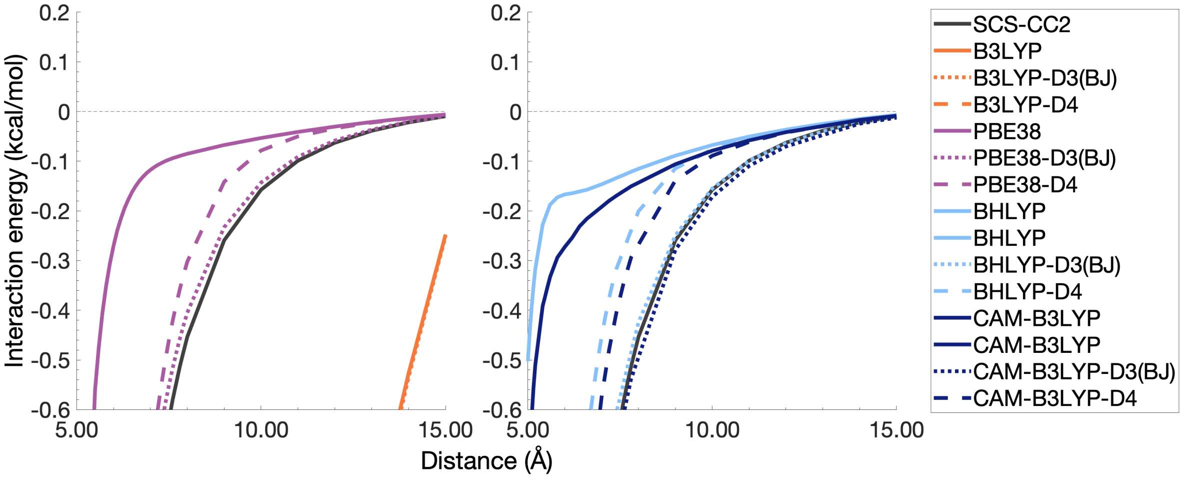

Figure S17: Mid-range region of the dissociation energy curves for the pyrene excimer comparing density functional approximations, with and without DFT-D type dispersion corrections, to the SCS-CC2/def2-TZVP reference. All TD-DFT results are based on the def2-TZVP basis set. As distances beyond the binding region were not the focus of the paper, some curves may show strange shapes in the mid-range due to the small number of data points however they still showcase the identified positive region.

SI.4.3 Numerical Data For Binding Minima and Associated Percentage errors

Table S5: Binding of the benzene excimer described by the dissociation energy (D_e; kcal/mol), equilibrium inter-monomer distance (r_e; Å) and the unsigned percentage errors of each compared to that of the SCS-CC2/CBS(3,4) reference.

	D_e			r_e		
Functional	Uncorrected	D3(BJ)	D4	Uncorrected	D3(BJ)	D4
B3LYP	10.68	21.24	21.47	3.15	3.03	3.03
PBE38	12.62	19.21	19.03	3.03	2.98	3.00
BHLYP	8.10	16.28	16.69	3.13	3.03	3.05
CAM-B3LYP	8.58	15.24	15.77	3.09	3.04	3.03
ωB97X[a]	11.99	-	-	3.08	-	-
B2PLYP	11.36	16.94	16.96	3.05	3.00	3.01
B2GP-PLYP	11.99	15.95	15.73	3.03	2.99	3.01
ωB2PLYP	13.07	13.20	13.19	2.99	2.99	2.99
ωB2GP-PLYP	13.90	13.90	13.90	2.98	2.98	2.98

	Percentage error in D_e			Percentage error in r_e		
Functional	Uncorrected	D3(BJ)	D4	Uncorrected	D3(BJ)	D4
B3LYP	21.7	55.5	57.3	6.1	2.0	2.0
PBE38	7.6	40.7	39.4	2.0	0.3	1.0
BHLYP	40.7	19.3	22.2	5.4	2.0	2.7
CAM-B3LYP	37.2	11.7	15.5	4.0	2.4	2.0
ωB97X[a]	12.2	-	-	3.7	-	-
B2PLYP	16.8	24.1	24.3	2.7	1.0	1.3
B2GP-PLYP	12.2	16.8	15.7	2.0	0.7	1.3
ωB2PLYP	4.2	3.3	3.4	0.7	0.7	0.7
ωB2GP-PLYP	1.8	1.8	1.8	0.3	0.3	0.3

[a] We assessed the pure ωB97X. Its various dispersion-corrected variants all depend on slightly different underlying XC expressions,[S1–S6] which is why dispersion-corrected results are not provided.

Table S6: Binding of the naphthalene excimer described by the dissociation energy (D_e; kcal/mol), equilibrium inter-monomer distance (r_e; Å) and the unsigned percentage errors of each compared to that of the SCS-CC2/CBS(3,4) reference.

	D_e			r_e		
Functional	Uncorrected	D3(BJ)	D4	Uncorrected	D3(BJ)	D4
B3LYP	14.69	30.94	31.54	3.37	3.16	3.16
PBE38	17.00	28.13	28.52	3.20	3.12	3.14
BHLYP	11.79	24.90	26.03	3.29	3.16	3.16
CAM-B3LYP	13.32	24.83	25.65	3.24	3.16	3.15
ωB97X[a]	18.95	-	-	3.24	-	-
B2PLYP	20.58	29.78	29.96	3.19	3.11	3.12
B2GP-PLYP	22.43	29.03	29.09	3.14	3.10	3.11
ωB2PLYP	22.72	22.98	22.99	3.09	3.09	3.09
ωB2GP-PLYP	24.67	24.68	24.68	3.07	3.07	3.07
	Percentage error in D_e			Percentage error in r_e		
Functional	Uncorrected	D3(BJ)	D4	Uncorrected	D3(BJ)	D4
B3LYP	48.1	9.3	11.4	10.9	3.9	3.9
PBE38	40.0	0.7	0.7	5.3	2.6	3.3
BHLYP	58.4	12.1	8.1	8.2	3.9	3.9
CAM-B3LYP	53.0	12.3	9.4	6.6	3.9	3.6
ωB97X[a]	33.1	-	-	6.6	-	-
B2PLYP	27.3	5.2	5.8	4.9	2.3	2.6
B2GP-PLYP	20.8	2.5	2.7	3.3	2.0	2.3
ωB2PLYP	19.8	18.8	18.8	1.6	1.6	1.6
ωB2GP-PLYP	12.9	12.9	12.9	1.0	1.0	1.0

[a] We assessed the pure ωB97X. Its various dispersion-corrected variants all depend on slightly different underlying XC expressions,[S1–S6] which is why dispersion-corrected results are not provided.

Table S7: Binding of the anthracene excimer described by the dissociation energy (D_e; kcal/mol), equilibrium inter-monomer distance (r_e; Å) and the unsigned percentage errors of each compared to that of the SCS-CC2/CBS(3,4) reference.

	D_e			r_e		
Functional	Uncorrected	D3(BJ)	D4	Uncorrected	D3(BJ)	D4
B3LYP	9.19	29.59	30.41	3.61	3.30	3.30
PBE38	11.31	26.05	27.12	3.40	3.28	3.30
BHLYP	6.88	23.40	25.17	3.51	3.31	3.31
CAM-B3LYP	8.18	23.50	24.44	3.45	3.32	3.30
ωB97X[a]	16.01	-	-	3.36	-	-
B2PLYP	17.23	29.24	29.55	3.36	3.25	3.26
B2GP-PLYP	19.82	28.44	28.78	3.31	3.24	3.25
ωB2PLYP	20.10	20.53	20.54	3.25	3.25	3.25
ωB2GP-PLYP	22.70	22.72	22.72	3.23	3.23	3.23
	Percentage error in D_e			Percentage error in r_e		
Functional	Uncorrected	D3(BJ)	D4	Uncorrected	D3(BJ)	D4
B3LYP	67.7	4.2	7.1	13.5	3.8	3.8
PBE38	60.2	8.3	4.5	6.9	3.1	3.77
BHLYP	75.8	17.6	11.4	10.4	4.1	4.1
CAM-B3LYP	71.2	17.2	14.0	8.5	4.4	3.8
ωB97X[a]	43.6	-	-	5.7	-	-
B2PLYP	39.3	3.0	4.1	5.7	2.2	2.5
B2GP-PLYP	30.2	0.1	1.3	4.1	1.9	2.2
ωB2PLYP	29.2	27.7	27.7	2.2	2.2	2.2
ωB2GP-PLYP	20.1	20.0	20.0	1.6	1.6	1.6

[a] We assessed the pure ωB97X. Its various dispersion-corrected variants all depend on slightly different underlying XC expressions,[S1–S6] which is why dispersion-corrected results are not provided.

Table S8: Binding of the pyrene excimer described by the dissociation energy (D_e; kcal/mol), equilibrium inter-monomer distance (r_e; Å) and the unsigned percentage errors of each compared to that of the SCS-CC2/CBS(3,4) reference.

	D_e			r_e		
Functional	Uncorrected	D3(BJ)	D4	Uncorrected	D3(BJ)	D4
B3LYP	10.60	33.41	34.37	3.67	3.31	3.31
PBE38	13.09	29.98	31.57	3.44	3.30	3.31
BHLYP	8.22	26.89	29.02	3.54	3.32	3.32
CAM-B3LYP	9.71	27.39	28.41	3.50	3.33	3.32
ωB97X[a]	17.97	-	-	3.37	-	-
B2PLYP	20.51	34.28	34.60	3.38	3.26	3.27
B2GP-PLYP	23.53	33.44	33.89	3.32	3.25	3.26
ωB2PLYP	23.11	23.63	23.66	3.27	3.27	3.27
ωB2GP-PLYP	26.29	26.32	26.32	3.25	3.25	3.25
	Percentage error in D_e			Percentage error in r_e		
Functional	Uncorrected	D3(BJ)	D4	Uncorrected	D3(BJ)	D4
B3LYP	68.2	0.4	3.2	15.0	3.8	3.8
PBE38	60.7	9.9	5.2	7.8	3.4	3.8
BHLYP	75.3	19.2	12.8	11.0	4.1	4.1
CAM-B3LYP	70.8	17.7	14.7	9.7	4.4	4.1
ωB97x	46.0	-	-	5.6	-	-
B2PLYP	38.4	3.0	3.9	6.0	2.2	2.5
B2GP-PLYP	29.3	0.5	1.8	4.1	1.9	2.2
ωB2PLYP	30.6	29.0	28.9	2.5	2.5	2.5
ωB2GP-PLYP	21.0	20.9	20.9	1.9	1.9	1.9

[a]We assessed the pure ωB97X. Its various dispersion-corrected variants all depend on slightly different underlying XC expressions,[S1–S6] which is why dispersion-corrected results are not provided.

SI.4.4 Mean Absolute Deviations

Table S9: Mean absolute deviations (MADs) for the dissociation energy (D_e; kcal/mol) and equilibrium distance (r_e; Å) characterising the description of excimer binding by the method relative to SCS-CC2/CBS(3,4) reference.

	D_e MADs			r_e MADs		
Functional	Uncorrected	DFT-D3(BJ)	DFT-D4	Uncorrected	DFT-D3(BJ)	DFT-D4
B3LYP	14.63	2.88	3.53	0.36	0.11	0.11
PBE38	12.41	2.85	2.15	0.17	0.07	0.09
BHLYP	17.17	4.36	3.21	0.27	0.11	0.12
CAM-B3LYP	15.97	3.97	3.41	0.23	0.12	0.11
ωB97x	9.69	-	-	0.17	-	-
B2PLYP	8.50	1.64	1.85	0.15	0.06	0.07
B2GP-PLYP	6.47	0.80	0.96	0.11	0.05	0.06
ωB2PLYP	6.16	5.83	5.82	0.06	0.06	0.06
ωB2GP-PLYP	4.15	4.14	4.14	0.04	0.04	0.04

[a]We assessed the pure ωB97X. Its various dispersion-corrected variants all depend on slightly different underlying XC expressions,[S1–S6] which is why dispersion-corrected results are not provided.

References

(S1) Chai, J.-D.; Head-Gordon, M. Systematic optimization of long-range corrected hybrid density functionals. *J. Chem. Phys.* **2008**, *128*, 084106.

(S2) Chai, J.-D.; Head-Gordon, M. *Phys. Chem. Chem. Phys.* **2008**, *10*, 6615–6620.

(S3) Lin, Y.-S.; Li, G.-D.; Mao, S.-P.; Chai, J.-D. Long-Range Corrected Hybrid Density Functionals with Improved Dispersion Corrections. *J. Chem. Theory Comput. Chem.* **2013**, *9*, 263–272.

(S4) Mardirossian, N.; Head-Gordon, M. ωB97X-V: A 10-parameter, range-separated hybrid, generalized gradient approximation density functional with nonlocal correlation, designed by a survival-of-the-fittest strategy. *Phys. Chem. Chem. Phys.* **2014**, *16*, 9904–9924.

(S5) Najibi, A.; Goerigk, L. The Nonlocal Kernel in van der Waals Density Functionals as an Additive Correction: An Extensive Analysis with Special Emphasis on the B97M-V and ωB97M-V Approaches. *J. Chem. Theory Comput.* **2018**, *14*, 5725–5738.

(S6) Najibi, A.; Goerigk, L. DFT-D4 counterparts of leading meta-generalized-gradient approximation and hybrid density functionals for energetics and geometries. *J. Comput. Chem.* **2020**, *41*, 2562–2572.
